# From Environmental Concentrations to Individual Inhalation: Analysis of Exposure Differences to PM_2.5_ and Chemical Components in Elderly Populations and Their Influencing Factors

**DOI:** 10.3390/toxics14050414

**Published:** 2026-05-10

**Authors:** Ruoyu Li, Fenghua Lin, Hao Zhang, Yuling Zhang, Shilin Chen, Dan Wang, Yongxin Wang, Haoneng Hu, Jianjun Xiang, Yu Jiang, Huaying Lin, Jianlin Zhu, Chuancheng Wu

**Affiliations:** 1 Department of Preventive Medicine, School of Public Health, Fujian Medical University, Fuzhou 350108, China; 18313150887@163.com (R.L.);; 2 The Key Laboratory of Environment and Health, School of Public Health, Fujian Medical University, Fuzhou 350108, China; 3 The Affiliated Fuzhou Center for Disease Control and Prevention of Fujian Medical University, Fuzhou 350209, China

**Keywords:** air pollution, PM_2.5_, chemical components, elderly population

## Abstract

(1) Background: This study investigated the characteristics and influencing factors of exposure to fine particulate matter (PM_2.5_) and its chemical composition among elderly residents, with the aim of revealing potential differences in exposure. (2) Methods: A total of 258 elderly individuals were monitored for 72 h through individual, indoor, and outdoor PM_2.5_ measurements. Concentrations were determined, and non-targeted components were analyzed by gas chromatography-mass spectrometry (GC-MS). Through Spearman correlation analysis, generalized linear model, and linear regression to explore the influencing factors. (3) Results: The individual PM_2.5_ concentration was higher than both the indoor and outdoor concentrations. A total of 20,962 compounds were detected in personal PM_2.5_ samples, 6794 in indoor PM_2.5_ samples, among which 4285 compounds were shared between the two sample types. The components were mainly esters, aromatic compounds, and amines. PM_2.5_ concentration was correlated with age, housing area, humidifier use, and second-hand smoke exposure. Chemical composition is related to outdoor pollution, furniture material, and daily behavior. (4) Conclusions: The individual PM_2.5_ concentration is higher than the environmental concentration, and its chemical composition overlaps with the indoor and outdoor environment, which is jointly affected by demography, living conditions, and daily behavior.

## 1. Introduction

Air pollution, particularly PM_2.5_ pollution, has emerged as a major global environmental and public health issue [1,2]. According to the World Health Organization, approximately 99% of the global population breathe air that exceeds the WHO air quality guideline limits. Ambient outdoor air pollution is estimated to have caused 4.2 million premature deaths worldwide in 2019, while the combined effects of ambient and household air pollution are associated with approximately 6.7 million premature deaths annually [3]. PM_2.5_ is recognized as a significant environmental risk factor and is closely linked to the incidence and mortality of diseases across multiple organ systems, especially the respiratory and circulatory systems [4,5,6]. Owing to its extremely small particle size, PM_2.5_ can travel long distances and carry toxic substances. It can penetrate the respiratory tract barrier, reach deep into the lungs, and even enter the bloodstream [7], resulting in serious health risks.

As a multi-source mixture, PM_2.5_ exhibits significant spatiotemporal variations in its chemical composition and toxicity, posing persistent and complex health risks to humans [8]. Studies have shown that even under exposure conditions below current regulatory limits, the association between PM_2.5_ and non-accidental deaths remains significant [9]. In so-called “low pollution” environments, the exposure-response curve in the low concentration range is steeper, which may be related to the higher toxicity of PM_2.5_ components in this range [10]. PM_2.5_ contains complex components such as polycyclic aromatic hydrocarbons, heavy metals, and organic compounds, which can trigger various chronic diseases through mechanisms like oxidative stress and inflammatory responses [11]. Moreover, the toxicity of different components varies significantly, offering new perspectives for further research on the health risks of PM_2.5_ [12,13].

Air pollution affects populations across all regions, age groups, and social classes, but certain subgroups, such as the elderly, children, and individuals with cardiovascular or respiratory diseases, are more vulnerable to the adverse health effects of air pollution [14,15]. Compared to younger individuals, the elderly are more sensitive to the effects of air pollution due to declines in physiological, metabolic, and compensatory functions, resulting in higher morbidity rates for related diseases [14,16]. With the rapid aging of the global population, reducing pollution exposure levels and improving the health of the elderly have become an urgent public health issue worldwide.

Current research primarily focuses on monitoring outdoor PM_2.5_ concentrations and assessing their health effects, overlooking the differences in indoor environments and individual exposure. In fact, individuals spend more than 80% of their time indoors, and the sources, chemical composition, and concentrations of indoor PM_2.5_ differ significantly from those of outdoor PM_2.5_ [17,18]. Personal exposure measurement using portable air pollution monitors is considered the gold standard for assessing human exposure to air pollution [19]. However, due to high costs and the heavy burden on participants, researchers often use convenient outdoor monitoring station concentrations or models as substitutes for actual personal pollution exposure indicators [20,21]. This may overlook differences in environments and individual behaviors, making it difficult to accurately reflect an individual’s true exposure levels, potentially leading to an overestimation or underestimation of health risks [22]. Therefore, personal monitoring provides more reliable data for accurate exposure assessment and health effect evaluation.

Individual exposure to PM_2.5_ and its associated chemical components is influenced by a combination of multiple factors. Total exposure results from the combined contributions of both environmental and non-environmental sources. Personal activity patterns and environmental factors significantly influence exposure levels, including indoor microenvironments, outdoor environments, daily activities, and physical condition [23,24]. In addition to outdoor sources such as coal combustion and vehicle exhaust, indoor sources of pollution, including smoking, cooking, and heating, also contribute significantly to total urban PM_2.5_ exposure; in some cases, depending on household characteristics, ventilation conditions, and seasonal factors, their contribution may approach or even equal that of outdoor sources [25].

Previous studies have shown that individual PM_2.5_ exposure does not fully align with indoor or outdoor fixed-point concentrations; this discrepancy is associated with the combined effects of indoor sources, outdoor infiltration, and daily activity patterns [26,27,28,29]. Studies among retired adults have found that 24-h personal PM_2.5_ concentrations are higher than indoor levels but lower than outdoor levels, and that factors such as indoor and outdoor PM_2.5_ levels, ventilation conditions, indoor pollution sources, and meteorological conditions all influence personal exposure [26]. A four-season study of the elderly in South Korea also showed that individual PM_2.5_ concentrations consistently exceeded indoor levels, while the relationship with outdoor concentrations was influenced by season and living environment [27]. Furthermore, a review study noted that there is significant variability between environmental PM_2.5_ and individual PM_2.5_ levels; using fixed-point environmental concentrations as a proxy for individual exposure may lead to misclassification of exposure. Smoking, cooking, heating, incense burning, traffic pollution infiltration, and ventilation conditions are key factors influencing indoor PM_2.5_ levels [28,29]. Therefore, comprehensive studies on individual, indoor, and outdoor PM_2.5_ exposure and its chemical characteristics in the elderly population remain necessary.

In recent years, with the development of exposomics and analytical technologies, researchers have been able to more systematically identify the harmful components of PM_2.5_ and their health effects [30]. A research team successfully identified hundreds of PM_2.5_ organic components associated with inflammatory responses using non-targeted exposomics techniques [24,31]. In addition, the application of artificial intelligence technologies has enabled high-resolution exposure assessment of ultrafine particles, providing new methods for the precise monitoring of PM_2.5_ chemical components [32]. These advancements have laid an important foundation for further elucidating the relationship between PM_2.5_ chemical components, individual exposure, and health effects.

Since PM_2.5_ is a complex mixture containing a variety of organic compounds, targeted analysis often covers only a limited subset of these chemicals. This study employed non-targeted GC-MS to screen a broader range of compounds and compare differences in chemical composition between individuals and indoor PM_2.5_ samples. However, this method has certain limitations: the identification of some compounds is provisional and requires further confirmation using reference standards, and the concentration results are primarily semi-quantitative. Given these characteristics, the untargeted GC-MS in this study was primarily used for chemical screening and compositional comparison, rather than for the definitive identification and absolute quantification of all compounds.

This study focuses on the elderly population in a community in Fuzhou, systematically assessing the exposure levels of PM_2.5_ and its chemical components based on simultaneous individual and indoor monitoring, and analyzing the impact of environmental factors and individual behaviors on PM_2.5_ exposure. The study aims to provide scientific evidence for accurately assessing health risks related to PM_2.5_ exposure in the elderly and to offer insights for developing personalized air pollution control strategies.

## 2. Materials and Methods

### 2.1. Study Subjects

We selected a community in Fuzhou for a field epidemiological investigation. A total of 258 study participants were recruited in July and August 2023 and 2024, and all participants signed informed consent forms. Sampling was limited to the same summer period over a two-year period to ensure comparability across seasons, minimize confounding effects arising from differences in temperature, humidity, ventilation patterns, and daily activities, and facilitate on-site implementation within the framework of the monitoring program. The study protocol was reviewed and approved by the Ethics Committee (Ethical Approval No. [2025] 60).

Inclusion criteria for study participants: Age ≥ 60 years, have resided in the community for more than 5 years, with no plans for travel in the past three months; regular daily routines, fixed dietary habits, and consistent activity patterns; no acute cardiovascular or respiratory events in the past three months.

### 2.2. Sample Collection and Measurement

#### 2.2.1. Criteria for Selecting Study Sites

**Figure 1:**
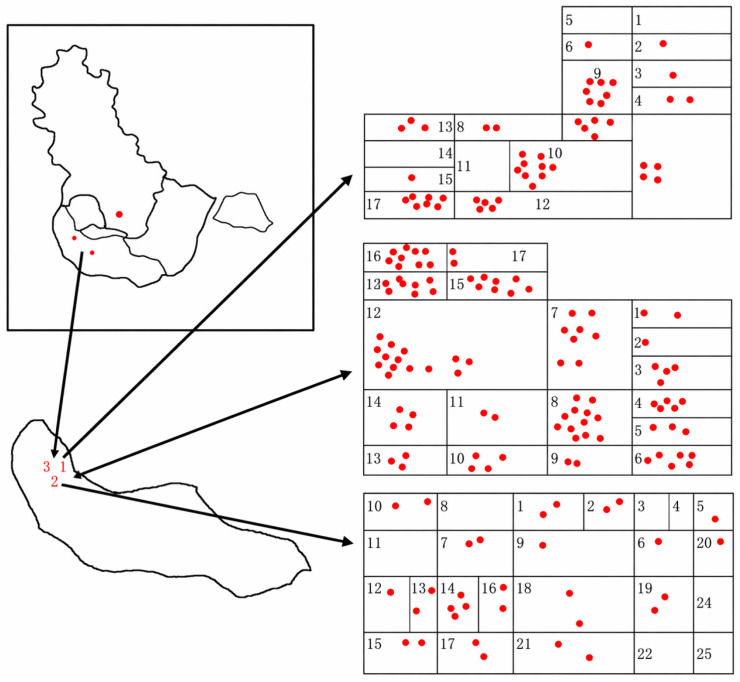
Selection of Study Sites and Distribution of Study Participants in Each Neighborhood. Note: Numbers represent community building identifiers, red dots represent study participants.

This study selected a community for on-site sampling in accordance with the national “Work Plan for Monitoring and Protection Against the Health Effects of Air Pollution (Haze).” The community meets the requirements of the “Technical Specifications for the Layout of Ambient Air Quality Monitoring Stations” (HJ 664—2013) [33] and is located approximately 1.5 km from a national PM_2.5_ monitoring station (within the representative range); It is situated at the boundary between two administrative districts, which helps minimize local anomalies; the community is well-equipped with infrastructure, contains no heavily polluting enterprises, and its PM_2.5_ concentration is generally comparable to the citywide average. The elderly residents in the community have typical lifestyles and demonstrate high levels of cooperation. Based on these conditions, this study selected three residential neighborhoods within the community for investigation (Figure 1).

#### 2.2.2. Sample Collection

Previous studies have found that significant adverse health effects in individuals are observed on the third day after PM_2.5_ exposure. Preliminary experimental results also indicate that a 3-day sampling period is sufficient to meet the detection limit requirements. Therefore, a three-day monitoring period was selected to assess environmental PM_2.5_ exposure.

Study participants wore the UPAS V2 personal exposure particulate matter sampler (Access Sensor Technologies, Fort Collins, CO, USA) for 72-h individual PM_2.5_ exposure monitoring (sampling for 24 h, followed by a 60-s pause), with a flow rate of 1 L/min for the personal sampler. The sampler was suspended at chest height within the breathing zone, and during rest periods, it was placed beside the bed to reflect the true exposure levels. After sampling, the individual sampling filters (PALL U.S., Port Washington, NY, USA) were collected, and the sampling volume and time were recorded. The filters were then stored at low temperatures and transported to the laboratory.

Indoor PM_2.5_ sampling was conducted using a temperature-controlled, constant-flow atmospheric particulate sampler (Model MH1205, Qingdao Minghua Electronic Instrument Co., Ltd., Qingdao, China). Continuous sampling was performed in the living room for 72 h with a flow rate of 40 L/min, while simultaneously monitoring the indoor microclimate. Considering that elderly individuals typically spend a significant amount of time in the living room and that this location is more convenient for implementation, it provides a good representation. Outdoor PM_2.5_ sampling for this study was conducted independently by the research team. Sampling points were established within national fixed monitoring stations, and the sampling periods were strictly synchronized with individual and indoor PM_2.5_ monitoring. The protocol was based on the indoor air quality standard (GB/T 18883-2002) [34].

#### 2.2.3. Participant Compliance

To ensure participant compliance during the 72-h monitoring period, field staff conducted daily home visits to check the operational status of individual UPAS V2 samplers and indoor samplers, including battery levels, flow rates, and any alarm notifications. In addition, multiple telephone follow-ups were conducted daily to remind participants to wear the samplers correctly and to record any abnormal activity logs. Any monitoring period with a valid sampling time of less than 80% will be excluded from the final analysis. In addition, the community has a collaborative relationship with the research team dating back more than a decade (since 2017), during which time they have completed numerous epidemiological surveys and environmental monitoring projects.

#### 2.2.4. Sample Analysis

The filter membrane treatment and weighing method were conducted according to the national “Air Pollution (Smog) Impact on Population Health Monitoring and Protection Program.” Before and after sampling, the quartz filter membranes were equilibrated in a temperature and humidity-controlled environment (25 ± 1 °C, 50 ± 5%) for 24 h. A balance with a sensitivity of 0.0001 g (METTLER TOLEDO, Greifensee, Switzerland) was used for weighing, with two measurements taken before and after sampling to obtain an average value. The PM_2.5_ mass concentration was calculated using the gravimetric method, with the following formula:

$$C=\frac{(W_{1}-W_{2})\times 10^{6}}{V_{0}}$$

*C* is the mass concentration (μg/m^3^), *W*_1_ and *W*_2_ are the masses of the filter membrane before and after sampling, respectively (g), and *V*_0_ is the sampling volume at standard conditions (m^3^).

#### 2.2.5. Sample Preparation

Using non-serrated forceps, fold the used filter membrane inward into a thin strip and place it in a 50 mL plastic centrifuge tube. Alternatively, cut the used filter membrane into small pieces and place them in an extraction flask. Add 10 mL of the extraction solvent (hexane-dichloromethane, 1:1, v/v) (Shanghai Titan Greagent, Shanghai, China) and sonicate at 40 °C for 20 min. Add another 10 mL of the extraction solvent and repeat the extraction once. Filter the extract into a brown glass tube and blow dry with nitrogen until nearly dry. Add 35 μL of a carbon tetrachloride (Shanghai Titan Adamas-beta, Shanghai, China)-acetonitrile (Merck Germany, Darmstadt, Germany) mixture, mix well, then add 5 mL of ultrapure water. Mix rapidly to form an emulsion, then let stand for 5 min. Centrifuge at 3500 rcf for 5 min, collect 200 μL of the supernatant, and store it in a sample vial for GC-MS (Agilent USA, Santa Clara, CA, USA) analysis.

#### 2.2.6. Procedure

The Agilent GC-MS was operated in high-resolution full-scan (SCAN) mode, with a mass-to-charge ratio (*m*/*z*) range of 50–650 amu. Concurrently, quality control (QC) samples and a standard sample (tetrabromobiphenyl ether, C_12_H_6_Br_4_O, at 1 mg/L) (Shanghai Zhenzhun Chemservice, Shanghai, China) were prepared. Using an autosampler or a sample loop, 5 μL of the standard solution and 5 μL of the actual sample are each dispensed. The samples are analyzed using a GC-MS instrument, and the retention times and peak areas of the chromatographic peaks are recorded. The concentrations of the various chemical components in the sample solution are calculated by normalizing the data based on the peak area of the standard solution. Normalize the peak area of the target compound against the peak area of C_12_H_6_Br_4_O to calculate the relative peak area (RPA): RPA = peak area of the compound/peak area of C_12_H_6_Br_4_O. Calculate the concentration of the target compound in the sample based on the sample’s RPA and the concentration of the standard; if the sample was diluted during extraction or dilution, the calculated result must be corrected.

The detected features were compared with blank samples, and background artifacts (such as peak drift) were removed. Features containing silicon were retained, but interpreted with caution.

We identified compounds by comparing mass spectra against the NIST20 spectral library; compounds with a match score of ≥90 were considered reliable results. This study employed a semi-quantitative method, using BDE-28 (1 mg/L) as an internal standard for relative peak area normalization. Unidentified compounds with a match score < 90 were discarded.

### 2.3. Questionnaire Survey

A standardized structured questionnaire was used, which included sections on individual PM_2.5_ exposure levels, general demographic characteristics, residential environment, health status information, individual behavioral factors, and physiological and biochemical indicators.

### 2.4. Quality Control

Investigators received uniform training, and samplers were calibrated and cross-checked for consistency before use. Parallel and blank samples were included in each batch. Laboratory testing was conducted using high-purity reagents and controlled by standard curves (correlation coefficient ≥ 0.999). Each batch of samples included blank and QC samples. Data review strictly followed the national protocol, and data below the detection limit were replaced with half of the detection limit. EpiData 3.1 software was used for double data entry and consistency validation of the questionnaires.

### 2.5. Statistical Methods

Statistical analyses were performed using SPSS 27 and R 4.3.0. Variables such as smoking, alcohol consumption, cooking behavior, fuel type, use of air purifiers, incense burning, and education level were categorized according to the study definitions. Other covariates included age, gender, BMI, time spent at home, and environmental temperature and humidity. Spearman correlation analysis was used to assess relationships among PM_2.5_ concentrations, chemical components, and meteorological or behavioral variables. First, univariate analyses were conducted to screen variables associated with indoor PM_2.5_, personal PM_2.5_, and selected chemical components. Variables with statistical significance were included in generalized linear models. These models identified factors associated with indoor and personal PM_2.5_ concentrations. For chemical component analyses, regression models evaluated associations between selected compounds and environmental, housing, or behavioral factors. Regression models also assessed how indoor environments influenced personal PM_2.5_ chemical exposure, with adjustments for relevant covariates. All statistical tests were two-tailed. *p* < 0.05 was considered statistically significant. The descriptive distribution of indoor and personal PM_2.5_ concentrations was visualized using box plots. Box plots were used to assess skewness and potential outliers. Outlier observations were retained if sampling records, instrument operation, and gravimetric results showed no obvious on-site or analytical errors.

## 3. Results

### 3.1. Basic Characteristics of Study Participants

A total of 258 elderly residents participated in the study, with an average age of 71.37 ± 7.07 years, including 114 males and 144 females (Table 1).

### 3.2. Exposure Characteristics and Determinants of Indoor PM_2.5_ and Its Chemical Components

#### 3.2.1. Exposure Characteristics of Indoor PM_2.5_ and Its Chemical Components

The average concentration of indoor PM_2.5_ across 84 samples was 7.67 µg/m^3^. The concentrations ranged from 1.959 to 25.150 µg/m^3^, with the maximum value approximately 12.84 times higher than the minimum. The average concentration of the chemical components was 12.54 ng/m^3^ (Table 2). (Only the top 40 compounds by concentration are presented here; concentrations of the remaining compounds are listed in Table S1; outliers have been removed; see the box plot in Figure S1).

#### 3.2.2. Correlation Analysis of Indoor PM_2.5_, Its Chemical Components, Individual Characteristics, and Meteorological Indicators

**Figure 2:**
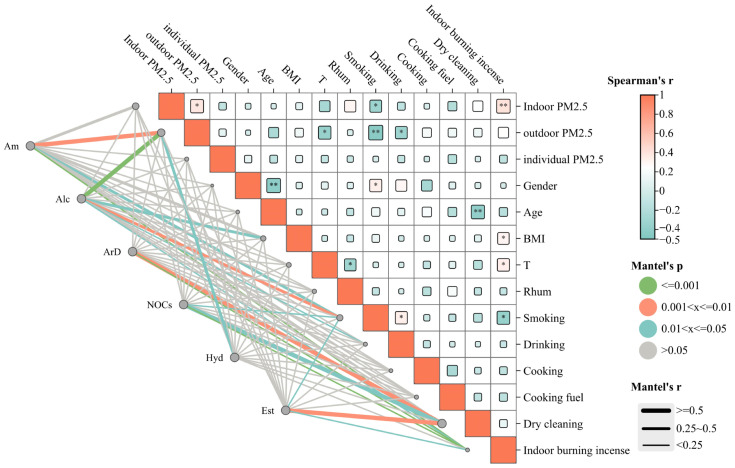
Spearman correlation analysis and Mantel test results for indoor PM_2.5_ concentrations, chemical components, meteorological indicators, and individual behaviors. Note: Spearman’s r is shown by the color scale, with darker colors indicating stronger correlations. Mantel’s *p* values are represented by line colors as follows: green, *p* ≤ 0.001; orange, 0.001 < *p* ≤ 0.01; blue, 0.01 < *p* ≤ 0.05; gray, *p* > 0.05. Am (Amine compounds), Alc (Alcohol compounds), ArD (Aromatic compounds), NOCs (Nitrogen-containing compounds), Hyd (Hydrocarbon compounds), and Est (Ester compounds).

A correlation analysis was conducted between indoor PM_2.5_ levels and their chemical components, individual characteristics (age, sex, and BMI), and environmental factors (temperature and humidity) (Figure 2). Indoor PM_2.5_ concentrations were positively correlated with outdoor levels and incense burning, but negatively associated with smoking behavior. Most compounds showed significant correlations with smoking and indoor incense burning, while amine compounds were strongly associated with outdoor PM_2.5_ concentrations. Alcohol compounds showed significant correlations with outdoor PM_2.5_ concentrations, BMI, and alcohol consumption behaviors. Aromatic compounds showed significant correlations with cleaning frequency. Nitrogen-containing compounds were also significantly associated with sweeping frequency. Hydrocarbons exhibited strong correlations with outdoor PM_2.5_ concentrations, while ester compounds were significantly associated with sweeping frequency.

#### 3.2.3. Influencing Factors of Indoor PM_2.5_ Exposure

Univariate analysis (Table A1) identified several factors significantly associated with indoor PM_2.5_ concentrations. Positive correlations were observed for outdoor PM_2.5_ levels (R = 0.441, *p* < 0.001), relative humidity (R = 0.298, *p* = 0.006), lower educational attainment (R = 0.378, *p* < 0.001), smaller housing area (R = 0.400, *p* < 0.001), daily time at home (R = 0.222, *p* = 0.042), frequent dry sweeping (R = 0.246, *p* = 0.024), incense burning (R = 0.409, *p* < 0.001), sofa usage over eight years (R = 0.231, *p* = 0.035), floor material (R = 0.334, *p* = 0.002), and daily exercise (R = 0.226, *p* = 0.039).

Negative correlations included ambient temperature (R = −0.272, *p* = 0.012), residence over ten years (R = −0.402, *p* < 0.001), smoking (R = −0.326, *p* = 0.002), secondhand smoke exposure (R = −0.219, *p* = 0.045), use of humidifiers (R = −0.258, *p* = 0.018), air fresheners or chemicals (R = −0.254, *p* = 0.020), and recent purchase of large furniture (R = −0.300, *p* = 0.006).

Other variables, including age, gender, BMI, floor level, proximity to major traffic arteries, drinking, cooking fuel, air purifier use, summer ventilation frequency, and daily air conditioning use, showed no statistically significant associations (*p* > 0.05).

Results from the multivariate generalized linear regression analysis (Table 3) showed that indoor PM_2.5_ concentrations were significantly associated with age (*p* = 0.001), housing area (*p* = 0.002), and the use of humidifiers (*p* = 0.028).

#### 3.2.4. Influencing Factors of Indoor Chemical Component Exposure

**Figure 3:**
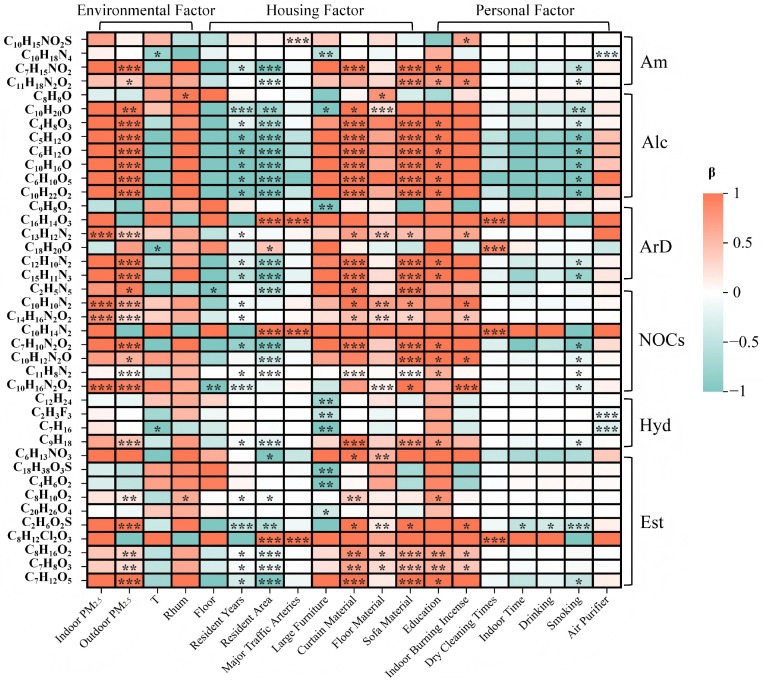
Heatmap of Regression Analysis Results for Indoor Compound Exposure and Influencing Factors (Top 40 Correlations). Note: The color in the heatmap represents the standardized regression coefficients (β values). * indicates statistical significance, with * *p* < 0.05, ** *p* < 0.01, and *** *p* < 0.001. Am (Amine compounds), Alc (Alcohol compounds), ArD (Aromatic compounds), NOCs (Nitrogen-containing compounds), Hyd (Hydrocarbon compounds), and Est (Ester compounds).

According to the univariate regression results of the top 40 compounds in indoor PM_2.5_ and their correlations with meteorological factors, housing conditions, individual characteristics, and behavioral activities (Figure 3), several compounds were found to be significantly correlated with these variables. Most compounds were positively correlated with outdoor PM_2.5_ concentrations, curtain and sofa materials, educational level, and indoor incense burning behavior, while they showed significant negative correlations with duration of residence, housing area, purchase of large furniture, flooring material, and smoking behavior.

Aromatic compound benzoylstyrene (C_16_H_14_O_3_), nitrogen-containing compound N-methylphenethylamine (C_10_H_14_N_2_), and ester compound dimethyl dichloroacetate (C_8_H_12_C_l2_O_3_) showed significant positive correlations with housing area, proximity to major traffic roads, and frequency of dry cleaning activities. Among the amine compounds, *p*-toluenesulfonamide (C_10_H_15_NO_2_S) showed a significant positive correlation with proximity to major traffic roads, while tetramethyl diamine (C_10_H_18_N_4_) was significantly negatively correlated with ambient temperature and the use of air purifiers. Among alcohol compounds, phenylethyl alcohol (C_8_H_8_O) showed a significant positive correlation with ambient humidity. Among aromatic compounds, benzothiazole (C_13_H_12_N_2_) showed a significant positive correlation with indoor PM_2.5_ concentrations, while styrenol (C_18_H_20_O) was negatively correlated with ambient temperature and significantly positively correlated with the frequency of dry cleaning. Among nitrogen-containing compounds, benzidine (C_10_H_10_N_2_) and diphenylamine (C_14_H_16_N_2_O_2_) showed significant positive correlations with indoor PM_2.5_ concentrations, while purine bases (C_2_H_5_N_5_) were significantly negatively correlated with residential floor level. Among hydrocarbon compounds, heptane (C_7_H_16_) showed a negative correlation with ambient temperature, while trifluoroethylene (C_2_H_3_F_3_) was significantly negatively correlated with the use of air purifiers.

### 3.3. Exposure Characteristics and Influencing Factors of Individual PM_2.5_ and Its Chemical Components

#### 3.3.1. Exposure Characteristics of Individual PM_2.5_ and Its Chemical Components

The average concentration of 258 individual PM_2.5_ samples was 38.06 µg/m^3^. The concentrations fluctuated within the range of 2.684 to 268.947 µg/m^3^. Among the chemical components, the ester compound dioctyl stearate (C_28_H_46_O_2_) had the highest concentration, with an average of 50.37 ng/m^3^ (Table 4). (Only the top 40 compounds by concentration are shown; the concentrations of the remaining compounds are provided in Supplementary Table S2; outliers have been removed; see the box plot in Figure S2).

#### 3.3.2. Correlation Analysis of Individual PM_2.5_ and Its Chemical Components, Individual Characteristics, and Meteorological Indicators

**Figure 4:**
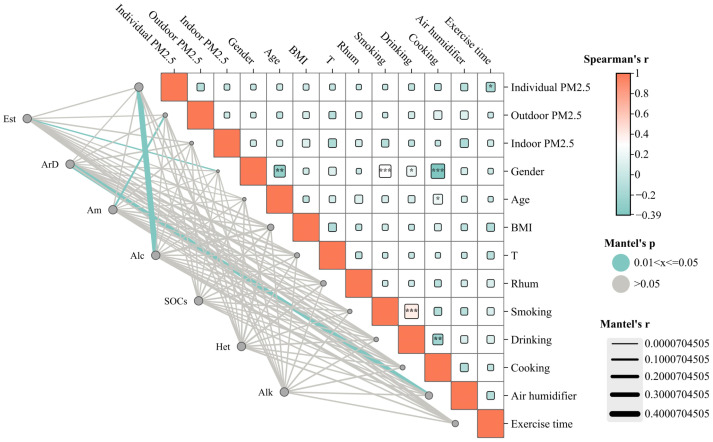
Spearman correlation matrix and Mantel test analysis of individual PM_2.5_, indoor PM_2.5_, outdoor PM_2.5_, chemical component subtypes, meteorological factors, and population behavioral characteristics. Note: Spearman’s r is shown by the color scale, with darker colors indicating stronger correlations. Mantel’s *p* values are represented by line colors as follows: blue, 0.01 < *p* ≤ 0.05; gray, *p* > 0.05. ArD (Aromatic compounds), Est (Ester compounds), Alk (Alkane compounds), Het (Heterocyclic compounds), Am (Amine compounds), SOCs (Silicon-containing organic compounds), and Alc (Alcohol compounds).

Correlation analysis was conducted between individual PM_2.5_ and its chemical components, individual characteristics (such as age, gender, BMI, and behavioral factors), and environmental factors (such as temperature and humidity) (Figure 4). Individual PM_2.5_ concentrations were negatively correlated with exercise duration. Individual PM_2.5_ concentrations showed no significant correlation with environmental factors or other general characteristics (such as age, gender, and BMI). Alcohols, amines, esters, and aromatic compounds were negatively correlated with individual PM_2.5_ concentrations, outdoor PM_2.5_ concentrations, gender, and the use of humidifiers, while no significant correlations were found with other factors.

#### 3.3.3. Influencing Factors of Individual PM_2.5_ Exposure

According to the univariate analysis results (Table A2), individual PM_2.5_ concentrations were positively correlated with the use of chemical products such as mosquito repellents and air fresheners. Individual PM_2.5_ concentrations were negatively correlated with the number of air conditioners (>2) and secondhand smoke exposure (yes). The multivariate generalized linear regression analysis results indicated a significant association between individual PM_2.5_ exposure and the duration of secondhand smoke exposure (Table 5).

#### 3.3.4. Influencing Factors of Individual Chemical Component Exposure

**Figure 5:**
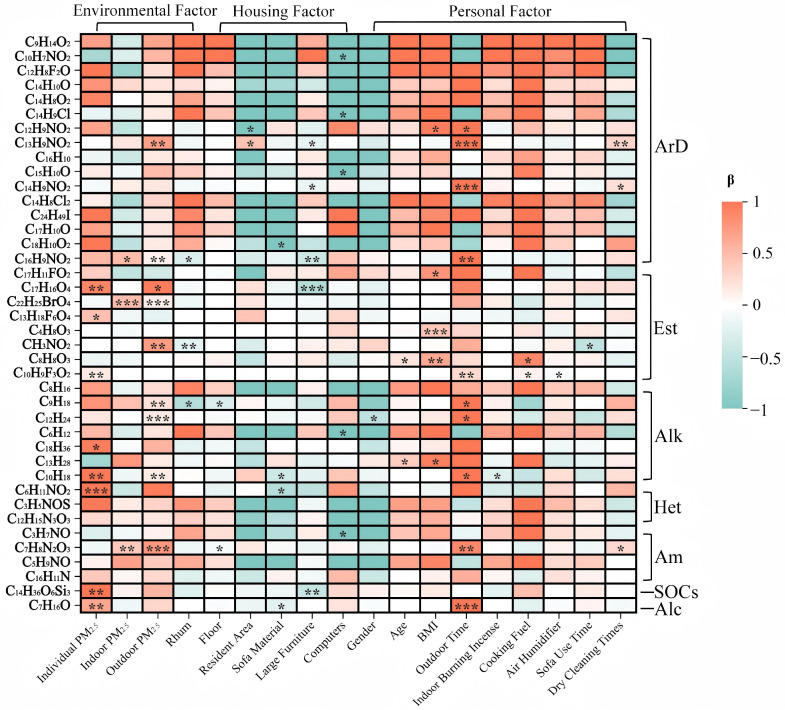
Heatmap of Regression Analysis Results for Individual Compound Exposure and Influencing Factors (Top 40 Correlations). * indicates statistical significance, with * *p* < 0.05, ** *p* < 0.01, and *** *p* < 0.001. Note: ArD (Aromatic compounds), Est (Ester compounds), Alk (Alkane compounds), Het (Heterocyclic compounds), Am (Amine compounds), SOCs (Silicon-containing organic compounds), and Alc (Alcohol compounds).

According to the correlation ranking, the univariate regression results for the top 40 compounds in individual PM_2.5_ and their correlations with meteorological factors, housing conditions, individual characteristics, and behavioral activities (Figure 5) indicate that several compounds are significantly correlated with these variables.

Among meteorological factors, most compounds (except for aromatic and amine compounds) showed positive correlations with individual PM_2.5_ concentrations, with the heterocyclic compound pyridin-3-one (C_6_H_11_NO_2_) being the most significant. Most compounds (except for heterocyclic, silicon-containing, and alcohol compounds) showed positive correlations with outdoor PM_2.5_ concentrations. Among them, diethyl oxalate (C_7_H_8_N_2_O_3_) showed the most significant correlation. Triphenylmethane bromide (C_22_H_25_BrO_4_), benzoylbenzimidazole (C_16_H_9_NO_2_), and diethyl oxalate (C_7_H_8_N_2_O_3_) showed positive correlations with indoor PM_2.5_ concentrations. Benzoylbenzimidazole (C_16_H_9_NO_2_), nitromethane (CH_3_NO_2_), and nonane (C_9_H_18_) showed negative correlations with ambient humidity.

Among housing factors, nonane (C_9_H_18_) and diethyl oxalate (C_7_H_8_N_2_O_3_) showed negative correlations with residential floor level. Phenylacetone (C_13_H_9_NO_2_) showed a positive correlation with duration of residence, while benzoic acid (C_12_H_9_NO_2_) showed a negative correlation. Phenylethanol (C_8_H_10_O_2_), nonane (C_10_H_18_), pyridin-3-one (C_6_H_11_NO_2_), and heptanol (C_7_H_16_O) showed negative correlations with sofa material. Phenylacetone (C_13_H_9_NO_2_), phenyl ethyl ketone (C_14_H_9_NO_2_), benzoylbenzimidazole (C_16_H_9_NO_2_), styrene (C_17_H_16_O_4_), and trimethoxypropylsilane (C_14_H_36_O_6_Si_3_) showed negative correlations with the purchase of large furniture, with styrene being the most significant. Cinnamic acid (C_10_H_7_NO_2_), chlorobenzene (C_14_H_9_Cl), benzyl alcohol (C_15_H_10_O), hexane (C_6_H_12_), and alanine (C_3_H_7_NO) showed negative correlations with the number of computers.

Among individual characteristics and behavioral factors, most compounds (except for heterocyclic and nitrogen-containing compounds) showed positive correlations with time spent outdoors, with aromatic compounds phenylacetone (C_13_H_9_NO_2_), phenyl ethyl ketone (C_14_H_9_NO_2_), and alcohol compound heptanol (C_7_H_16_O) being the most significant. Dodecane (C_12_H_24_) showed a negative correlation with gender, while methyl benzoate (C_8_H_8_O_3_) and nonane (C_10_H_18_) were positively correlated with age. The alkane compound nonane (C_10_H_18_) showed a negative correlation with indoor incense burning behavior. Ester compounds methyl benzoate (C_8_H_8_O_3_) and trifluorochlorophenylacetic acid (C_10_H_9_F_3_O_2_) were positively correlated with the type of cooking fuel used. Trifluorochlorophenylacetic acid (C_10_H_9_F_3_O_2_) showed a negative correlation with the use of indoor humidifiers. Nitromethane (CH_3_NO_2_) was negatively correlated with sofa usage time. Aromatic compounds phenylacetone (C_13_H_9_NO_2_), phenyl ethyl ketone (C_14_H_9_NO_2_), and diethyl oxalate (C_7_H_8_N_2_O_3_) showed positive correlations with the frequency of dry cleaning. The results indicate that different compounds in individual PM_2.5_ are influenced by multiple interacting factors. There are distinct differences in the sources, transmission, and accumulation mechanisms of various compound categories.

#### 3.3.5. Comparison of Individual, Indoor, and Outdoor PM_2.5_ Concentrations

A comparative analysis was conducted to statistically verify whether there were differences between individual PM_2.5_ exposure levels and environmental monitoring levels. The Wilcoxon signed-rank test was used to compare individual and indoor PM_2.5_ concentrations. The results showed that individual PM_2.5_ levels were significantly higher than indoor levels (positive ranks: *n* = 215, median rank = 144.11; negative ranks: *n* = 43, mean rank = 56.44; Z = −11.901, *p* < 0.001). For the comparison between individual and outdoor PM_2.5_ concentrations, a one-sample *t*-test was performed on the difference (individual–outdoor). The results showed a statistically significant difference (*p* = 0.01), with the mean individual PM_2.5_ concentration significantly higher than the outdoor concentration. This highlights the necessity of conducting individual exposure assessments beyond traditional environmental monitoring (Supplementary Tables S3 and S4).

### 3.4. Exposure Characteristics of Chemical Components in Environmental and Individual PM_2.5_

**Figure 6:**
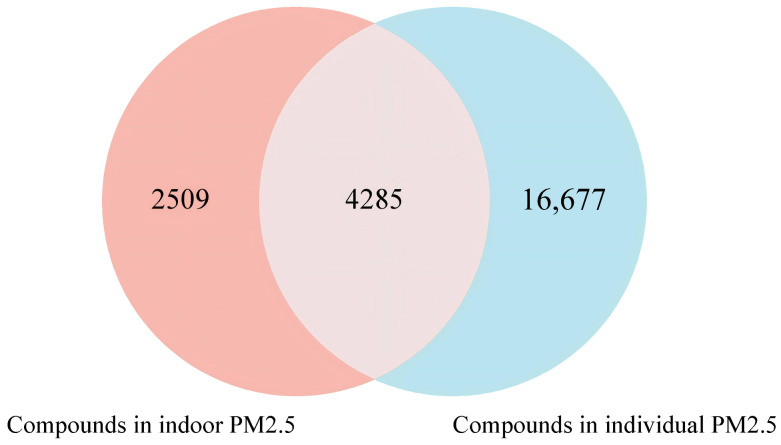
Wayne diagram of intersections of indoor and individual PM_2.5_ chemical components. Note: A total of 4285 compounds were shared between indoor and personal PM_2.5_ samples, accounting for 63.1% of the total compounds detected in indoor samples (6794) and 20.4% of those detected in personal samples (20,962).

After excluding background compounds such as column bleed and instrumental noise, 6794 compounds were identified in the 84 indoor PM_2.5_ filters, while 20,962 compounds were identified in the 258 individual PM_2.5_ filters. A total of 4285 common compounds were detected in both environmental and individual samples, accounting for 63.07% of the total indoor compounds and 20.44% of the total individual compounds (Figure 6).

**Figure 7:**
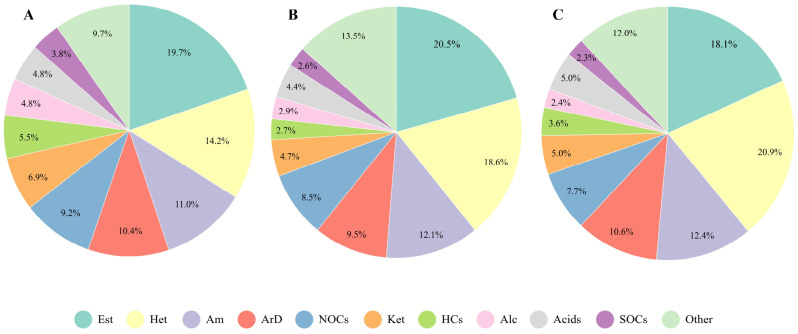
Composition of Chemical Components in Individual and Indoor PM_2.5._ (**A**) Composition of chemical components in individual PM_2.5_; (**B**) Composition of chemical components in indoor PM_2.5_; (**C**) Composition of shared chemical components in both indoor and individual PM_2.5_. Note: Est (Ester compounds), Het (Heterocyclic compounds), Am (Amine compounds), ArD (Aromatic compounds), NOCs (Nitrogen-containing organic compounds), Ket (Ketone compounds), HCs (Hydrocarbon compounds), Alc (Alcohol compounds), Acids (Acid compounds), SOCs (Silicon-containing organic compounds), and Other (Other compounds).

The composition analysis revealed that the chemical components in individual and indoor PM_2.5_ samples were similar, with the predominant compounds being esters, heterocyclic compounds, amines, aromatic hydrocarbons, and their derivatives. These compounds were highly prevalent in both indoor and individual exposure (Figure 7). Moreover, while there was some overlap between the chemical components of indoor PM_2.5_ and individual exposure, differences were also observed, suggesting that the compounds in individual PM_2.5_ not only originate from the indoor environment but are also influenced by behavioral activities, living habits, and other factors.

### 3.5. Correlation Analysis of Shared Chemical Components in Environmental and Individual PM_2.5_

**Figure 8:**
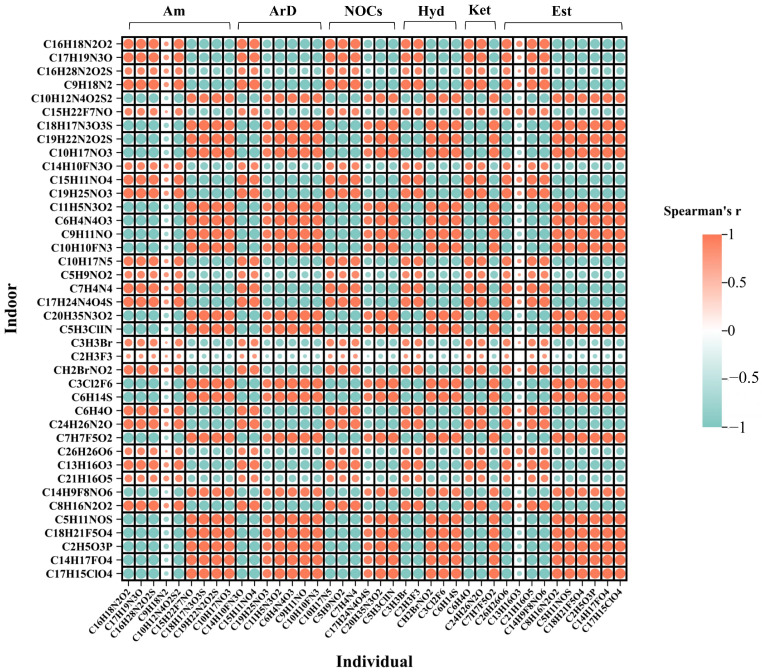
Correlation Heatmap of Shared Chemical Components in Environmental and Individual PM_2.5_ (Top 40 Listed Only). Note: The larger the circle, the stronger the correlation; Est (Ester compounds), Am (Amine compounds), ArD (Aromatic compounds), NOCs (Nitrogen-containing organic compounds), Ket (Ketone compounds), and Hyd (Hydrocarbon compounds).

Based on the correlation heatmap of shared exposure between indoor and individual PM_2.5_ (Figure 8), there is a complex interplay between the chemical components of PM_2.5_, with both positive and negative correlations observed. The correlation analysis revealed distinct clustering patterns, indicating that certain PM_2.5_ components exhibit strong correlations in both indoor and individual exposures, suggesting that they may share similar sources.

This suggests that these compounds are ubiquitous pollutants in the environment, or that individuals primarily engage in indoor activities, leading to a high consistency between their exposure levels and indoor pollutant concentrations. However, the observed differences between indoor and individual exposures may be influenced by various factors, including different exposure sources and individual behavior patterns. The results suggest that when studying PM_2.5_ exposure, it is crucial to consider the complex interactions between the environment and individual behaviors to more accurately assess actual exposure levels in populations.

### 3.6. Influencing Factors of Shared Exposure to Environmental and Individual PM_2.5_

Building on the analysis of the individual influencing factors, a further adjustment was made for covariates such as age, gender, BMI, smoking, alcohol consumption, and cooking. Univariate analysis was then performed on the compounds shared between indoor and individual exposure (Table A3). Several chemical components showed significant correlations. The amine compound phenylsulfonamide (C_18_H_17_N_3_O_3_S) exhibited a significant correlation between individual and indoor exposures, suggesting that its distribution varies across different exposure media. The ester compound methyl eugenol (C_21_H_16_O_5_) was also a major differentiating compound, indicating a significant difference in its concentration levels between individual exposure and the indoor environment.

Overall, most chemical components showed small differences between the indoor environment and individual exposure, with high *p*-values. This suggests that these compounds may have similar sources or that they are closely related to the fact that individuals primarily engage in indoor activities. The univariate analysis further revealed that only a few chemical components showed significant differences between individual and indoor exposures. Most components exhibited high consistency, indicating that indoor pollution plays a significant role in determining individual exposure levels.

## 4. Discussion

This study employed individualized particulate matter samplers and portable air pollution monitors to assess the indoor and personal PM_2.5_ exposure levels among elderly residents in a community in Fuzhou. Overall, individual PM_2.5_ exposure levels were higher than the corresponding indoor and outdoor concentrations, and the chemical composition of individual samples showed some overlap with that of indoor samples but was not entirely consistent. These findings suggest that the actual exposure of older adults is influenced not only by background environmental concentrations but also by the combined effects of various microenvironments, daily activity patterns, and exposure to local sources. However, it should be noted that all sampling in this study was conducted during the summer (July–August); therefore, the results and related discussions primarily reflect summer characteristics and cannot be directly extrapolated to other seasons.

Research has found that individual PM_2.5_ exposure levels cannot be fully represented by fixed indoor or outdoor monitoring points. Fixed-point monitoring primarily reflects the average pollution level at a specific spatial location, whereas personal monitoring, situated within or close to the breathing zone, is better able to capture the comprehensive exposure experienced by study participants in real-life situations [35]. For older adults, although their daily activity patterns are relatively stable and they spend most of their time indoors, their exposure is not limited to fixed sampling points in the living room. Activities such as cooking, cleaning, burning incense, using household chemicals, being near the kitchen or windows, moving between the bedroom and living room, brief outings, and entering stairwells or communal spaces within the neighbourhood may all result in short-term high-exposure events. Such near-source exposure peaks are more readily captured by personal samplers, whereas fixed-point monitoring, indoors or outdoors, may underestimate actual inhalation exposure levels due to fixed sampling locations, spatial dilution, or temporal averaging [36]. Consequently, even where ambient air quality at the community level is generally good, individual exposure may still be relatively high.

Higher individual exposure levels may also be linked to the resuspension of settled particulate matter indoors. When elderly residents walk around, clean, make their beds, or handle furniture and clothing, particulate matter settled on the floor, bedding, sofas, and clothing surfaces can be released back into the air, leading to increased particulate matter concentrations in the breathing zone [37]. As older adults spend more time at home and their activities tend to take place within relatively fixed indoor spaces, this local resuspension process may further amplify the discrepancy between individual exposure and fixed-point indoor concentrations. Consequently, individual PM_2.5_ exposure is more likely to result from the combined effects of indoor background pollution, emissions from nearby sources, resuspended particulate matter, and personal activity patterns, rather than being a direct reflection of a single fixed-point indoor concentration [38].

Chemical composition analysis further supports the above conclusion. Individual PM_2.5_ particles consist primarily of organic compounds such as esters, aromatic compounds, and amines, whilst aromatic compounds and esters also account for a significant proportion of indoor samples. There is a degree of overlap in the chemical composition between the two types of samples, indicating that the indoor environment is one of the major sources of individual PM_2.5_ exposure among the elderly; however, the two are not entirely consistent, suggesting that individual exposure cannot simply be regarded as a direct substitute for indoor air composition. Rather, the chemical composition of individual PM_2.5_ is more likely to be a composite result arising from the combined effects of indoor emissions, the infiltration of outdoor pollutants, individual activities, and contact with local sources.

In indoor PM_2.5_ samples, aromatic and ester compounds were relatively prominent. Chemical signatures represented by ethyl p-hydroxybenzoate (C_8_H_9_NO_2_), diethyl dodecylate (C_12_H_22_O_7_), and octanol (C_8_H_18_O), among others, may reflect the combined contribution of indoor consumer products, building materials, and daily activities. As an aromatic compound, ethyl benzoate may be associated with volatile and semi-volatile organic compounds released from air fresheners, perfumes, cleaning products, paints, or building and decorative materials [39]. Such aromatic compounds can undergo partitioning between indoor air, particulate matter, and surface materials, and adsorb onto the surface of PM_2.5_ to form particulate-bound exposure. As an ester compound, diethyl dodecylate may be associated with the heating of fats and oils during cooking, oxidation products of vegetable oils or animal fats, flavouring additives, and emissions from plastic and rubber materials [40]. Cooking fumes often contain various fatty acid esters, aldehydes, ketones, and oxidation products; these substances can enter the particulate phase through condensation, adsorption, or secondary reactions [41,42]. Octanol, as an alcohol compound, may originate from the volatilisation of solvents in paints, inks, adhesives, cleaning agents, and household products; its detection in indoor PM_2.5_ suggests that household products and building materials may be significant sources of indoor organic compounds.

The association between indoor PM_2.5_ and its chemical composition, on the one hand, and meteorological factors, housing conditions, and behavioural activities, on the other, is also of considerable environmental significance [43,44]. Higher outdoor PM_2.5_ concentrations may enter indoor spaces via ventilation and air exchange, representing a significant external source of indoor pollution. Higher humidity may increase the hygroscopicity of particulate matter, affecting its settling, residence time, and the partitioning of semi-volatile organic compounds between the gas and particulate phases. The burning of incense and frequent cleaning indoors can increase indoor PM_2.5_ and particle-bound organic matter levels through the direct release of particulate matter and the disturbance of settled dust, respectively. Multivariate analysis suggests that age, living space, length of residence, and the use of humidifiers are associated with indoor PM_2.5_ exposure during the summer [45]. Older individuals tend to have a relatively stable range of activity and spend more time indoors, making them more susceptible to the accumulation and resuspension of pollutants in the home environment. Smaller living spaces may result in a smaller volume for pollutant dilution, higher source density, and poorer diffusion capacity, thereby increasing exposure levels. The use of humidifiers may influence the processes of hygroscopic growth, settling, and resuspension of particulate matter by altering indoor humidity; however, it may also reflect differences in household air management practices, and therefore, its role requires further research to verify.

In terms of specific chemical components, nitrogen-containing compounds such as biphenylamine and diphenylamine showed a positive correlation with indoor PM_2.5_ levels, suggesting a possible link to indoor combustion, cooking, tobacco residues, or secondary reactions involving nitrogen-containing precursors [46]. Most chemical constituents showed a positive correlation with outdoor PM_2.5_ concentrations, suggesting that outdoor pollution sources, such as traffic emissions, may enter the indoor environment via ventilation, infiltration through windows and doors, or air exchange [47,48]. Ambient temperature showed a negative correlation with some compounds, which may be related to higher temperatures promoting volatilisation, desorption, or chemical transformation; whereas the correlation between phenethyl alcohol and ethyl benzoate with relative humidity may reflect the regulatory role of humidity in the hygroscopic growth of particulate matter and the gas-particle partitioning of organic compounds [49]. Housing conditions and household behaviours can also alter the sources and distribution of indoor chemical constituents. For example, tiled floors are more likely to disturb settled dust during cleaning, leading to the resuspension of particulate matter; homes located near major roads are more susceptible to the infiltration of vehicle exhaust and road dust; the burning of incense indoors can directly release particulate matter and various organic compounds; and air purifiers may reduce certain particulate matter and their bound chemical compounds through filtration [50,51]. These findings suggest that the chemical composition of indoor PM_2.5_ is not determined by a single source, but rather results from the combined effects of outdoor infiltration, indoor emissions, material releases, and daily activities.

In individual PM_2.5_ samples, components such as esters, aromatic hydrocarbons and their derivatives, amines, and organosilicon compounds are relatively prominent. Dioctyl stearate (C_28_H_46_O_2_) may be associated with sources such as lipid degradation, lubricants, plastic additives, or surface coatings [52]. Due to its high hydrophobicity, it readily adsorbs onto the surface of fine particulate matter and is resuspended into the respiratory tract via clothing, furniture, plastic products, or settled dust. Diisooctyl phthalate (C_13_H_18_O_3_) may indicate exposure to plastic products, soft materials, flooring, wallpaper, furniture, or consumer goods containing plasticisers. This class of semi-volatile organic compounds can be continuously released from materials and is more readily captured in personal breath samples. Triphenylsilane (C_17_H_16_O_3_Si) and other organosilicon compounds may originate from silicone-based personal care products, sealants, lubricants, furniture surface treatments, or household materials [53], and may also be partly attributable to the analytical background. Therefore, the interpretation of organosilicon compounds in non-targeted GC-MS results should be approached with caution, taking into account blank samples and quality control results.

Aromatic hydrocarbon derivatives and nitrogen-containing organic compounds in individual samples also provide clues as to sources of pollution. Such compounds may be associated with aromatic solvents, combustion emissions, infiltration of traffic pollution, cleaning products, flame retardants, fluorinated or halogenated consumer goods, and emissions from indoor materials. Aromatic compounds typically exhibit high environmental persistence and a strong affinity for particulate matter; they can enter indoor environments via infiltration from outdoor pollution [28] or be released from indoor sources such as incense burning, cooking fumes, building materials, or cleaning products. Amines and other nitrogen-containing organic compounds may be associated with cooking, tobacco residues, cleaning products, indoor biogenic sources, or secondary reactions involving nitrogen-containing precursors [54].

Differences between indoor and personal PM_2.5_ chemical compositions may also be influenced by indoor physicochemical processes. Many bound organic compounds in PM_2.5_ are semi-volatile and can undergo dynamic exchange between the air, particulate matter, and indoor surfaces. Rising temperatures may promote volatilisation, desorption, or chemical transformation; changes in humidity, in turn, can affect the hygroscopic growth, settling, and resuspension of particulate matter, as well as the gas-particle partitioning of polar organic compounds. Indoor surfaces such as walls, furniture, curtains, carpets, clothing, and bedding can act as ‘reservoirs’ for organic chemicals, adsorbing pollutants under certain conditions and re-releasing them when disturbed by daily activities, thereby prolonging the residence time of pollutants in the indoor environment. Activities such as cleaning, walking, sitting, or lying on sofas, or changing bedding, may all promote the resuspension of settled particulate matter and the chemical constituents it carries. Consequently, the observed differences in chemical composition reflect not only distinct sources of pollution but also the complex dynamic exchange processes between the air, particulate matter, indoor surfaces, and human activities.

Individual exposure to PM_2.5_ and its chemical composition is associated with a variety of residential and behavioural factors. Older adults spend more time indoors and have a relatively stable range of activities; consequently, they are more susceptible to the influence of the domestic microenvironment and localised sources of pollution. Smaller living spaces may hinder the dispersion of pollutants, whilst furniture, flooring materials, curtains, sofas, and household items can alter the chemical composition of indoor PM_2.5_ by releasing, adsorbing, or re-releasing pollutants. Burning incense and cooking can directly release particulate matter and organic compounds; cleaning may disturb settled dust and lead to the resuspension of adsorbed pollutants; air purifiers may reduce some particulate matter and its bound chemical compounds through filtration; and the use of air conditioning may influence individual exposure by altering air circulation, filtration status, window-opening behaviour, and indoor thermal comfort conditions. The association between humidifier use and PM_2.5_ levels may reflect the influence of humidity on particle deposition and hygroscopic growth, or it may reflect differences in air management practices among households that use humidifiers [55]. Consequently, PM_2.5_ exposure among older adults is not determined by a single source, but rather results from the interaction of the built environment, household behaviours, and individual activities.

It should be noted that some of the associations identified in this study should be interpreted with caution. The negative associations observed between smoking-related variables, second-hand smoke exposure, or certain household chemicals and PM_2.5_ do not fully align with existing knowledge and cannot be interpreted as indicating a genuine “reducing effect”. Although the time-activity patterns suggest that smokers typically take measures such as opening windows for ventilation, leading to lower indoor PM_2.5_ concentrations during the sampling period. These inverse associations may be related to differences in air exchange rates, mismatches between the timing of pollutant source activity and the monitoring period, exposure misclassification, or residual confounding. At the same time, a small number of amines and nitrogen-containing compounds showed a positive correlation with the duration of second-hand smoke exposure, suggesting that residual and re-released pollutants from second-hand and third-hand smoke may still result in long-term low-dose exposure [56]. Therefore, the above results are better regarded as exploratory findings rather than causal explanations.

In this study, a number of common chemical constituents were detected in both indoor and individual samples, primarily comprising esters, heterocyclic compounds, amines, aromatic hydrocarbons, and their derivatives [57]. This indicates that the indoor environment makes a significant contribution to individual exposure. However, differences in the concentrations of aromatic derivatives, esters, and amines between indoor and individual PM_2.5_ samples also suggest that individual exposure is influenced by factors such as frequency of outdoor activities, localised pollution sources, physical activity habits, smoking, diet, and the use of household chemicals [58,59]. Differences in certain compounds may reflect behavioural sources, such as smoking, the use of chemical products, air fresheners, gas appliances, and cleaning activities [60,61]; whereas differences in other compounds are smaller, possibly because their widespread presence in indoor environments results in relatively similar exposure levels across different individuals.

By simultaneously monitoring individual, indoor, and outdoor PM_2.5_ concentrations and chemical compositions, this study provides Supplementary Information for existing exposure assessment studies. Unlike most previous studies, which relied solely on outdoor monitoring data or single-site indoor environmental monitoring, this study employs a multi-scenario, simultaneous, and continuous monitoring strategy, which helps to provide a more comprehensive understanding of the actual PM_2.5_ exposure patterns among the elderly. The results suggest that using indoor or outdoor concentrations alone to estimate individual exposure may introduce certain biases. This finding can serve as a reference for optimizing future exposure assessment models and refining health risk assessment methods, thereby advancing research in the field of population exposure assessment.

This study has several limitations: it employed a cross-sectional design, which precludes the establishment of causal relationships; the study sample was limited to a single community in Fuzhou, introducing selection bias and limiting the generalizability of the results; and monitoring was confined to the summer months, failing to account for the impact of seasonal environmental variations and lifestyle differences on PM_2.5_ concentrations and composition. Additionally, the qualitative analysis of chemical composition was largely preliminary, and the quantitative results were only semi-quantitative. The study did not correct for intra-household correlation using a clustering model, which may introduce statistical limitations. Future research could expand the study scope, conduct cross-seasonal monitoring, and incorporate targeted testing to further refine the exposure assessment system.

## 5. Conclusions

There are discrepancies between actual individual PM_2.5_ exposure levels and their chemical composition, and the results of indoor environmental monitoring. Even when both outdoor and indoor air quality meet current standards, individuals may still be at risk of air pollution due to indoor emission sources or personal behaviors. Exposure to indoor and individual PM_2.5_ and its chemical composition is influenced by a combination of meteorological conditions, housing characteristics, and personal behaviors.

These findings suggest that reliance on fixed-point monitoring in epidemiological studies may lead to exposure misclassification bias, thereby underestimating health effects or masking associations with specific components. Future studies should incorporate personal monitoring to improve exposure assessment. Furthermore, since older adults and vulnerable populations spend more time indoors and have higher susceptibility, their exposure risks are particularly pronounced; personal monitoring can effectively identify their exposure hotspots and behavioral factors. Finally, current compliance-based air quality standards may create the misleading impression that “meeting standards equals safety,” and there should be a shift toward health-oriented personal exposure management. Future research should further compare different exposure scenarios, refine assessment methods, and explore the interaction mechanisms among multiple pollutants to support the development of targeted interventions and precise public health strategies.

## Figures and Tables

**Table 1:** Basic Characteristics of Study Participants.

Characteristics	Value
Continuous variables, mean ± SD	
Age	71.37 ± 7.07
BMI	24.11 ± 3.01
Indoor Time (Hour)	20.98 ± 2.71
Outdoor Time (Hour)	3.01 ± 2.71
Categorical variables, N (%)	
Gender	
Male	114 (44.19)
Female	144 (55.81)
Education	
Below Primary or Above Undergraduate	28 (10.85)
Regular Primary Schools	48 (18.60)
Junior Secondary Schools	66 (25.58)
Senior Secondary Education	60 (23.26)
Professional College	56 (21.71)
Smoking	
No	227 (87.98)
Yes	31 (12.02)
Passive smoking	
No	194 (75.19)
Yes	64 (24.81)
Passive smoking time per day (Hour)	
<0.5	226 (87.60)
0.5–1	16 (6.20)
>1	16 (6.20)
Drinking	
No	214 (82.95)
Yes	44 (17.05)
Cooking	
No	81 (31.40)
Yes	177 (68.60)
Exercises per day (Hour)	
<0.5	44 (17.06)
0.5–1	91 (35.27)
>1	123 (47.67)
Residence years (Year)	
≤10	72 (27.91)
>10	186 (72.09)
Indoor burning incense	
No	140 (54.26)
Yes	118 (45.74)

**Table 2:** Basic characteristics of chemical components in indoor PM_2.5_.

	N	Mean ± SD	Minimum	Maximum	IQR
T (°C)	84	29.26 ± 2.87	21.700	34.500	3.200
Rhum (%)	84	73.23 ± 11.35	40.300	90.730	14.870
Indoor PM_2.5_ (μg/m^3^)	84	7.67 ± 5.28	1.959	25.150	5.686
Aromatic hydrocarbon derivatives (ArD)					
C_8_H_9_NO_2_ (ng/m^3^)	84	12.54 ± 56.40	1.205	370.861	5.059
C_12_H_14_O_6_ (ng/m^3^)	84	2.25 ± 1.61	1.331	6.708	1.494
C_18_H_23_N_3_ (ng/m^3^)	84	1.64 ± 7.72	0.182	50.727	0.663
C_14_H_22_O_2_ (ng/m^3^)	84	0.79 ± 2.53	0.068	16.494	0.541
C_8_H_13_N_7_ (ng/m^3^)	84	0.78 ± 2.81	0.023	18.383	0.456
C_13_H_23_F_3_N_2_O_2_ (ng/m^3^)	84	0.70 ± 2.29	0.042	13.960	0.275
C_11_H_11_BrO_4_ (ng/m^3^)	84	0.66 ± 0.61	0.114	2.038	0.837
C_13_H_13_F_5_O_2_ (ng/m^3^)	84	0.65 ± 2.60	0.095	17.135	0.33
C_6_H_5_N_7_O_2_ (ng/m^3^)	84	0.62 ± 0.63	0.099	2.371	0.936
C_14_H_6_F_10_O_5_ (ng/m^3^)	84	0.58 ± 2.32	0.093	15.308	0.294
C_12_H_16_N_2_O_3_ (ng/m^3^)	84	0.51 ± 0.64	0.074	2.939	0.485
C_15_H_11_N_3_O_3_ (ng/m^3^)	84	0.49 ± 1.98	0.097	13.010	0.101
C_13_H_9_ClIN_3_O (ng/m^3^)	84	0.44 ± 2.19	0.050	14.339	0.013
C_11_H_14_N_2_OS (ng/m^3^)	84	0.42 ± 0.54	0.094	1.747	0.614
C_10_H_7_F_2_N_3_ (ng/m^3^)	84	0.30 ± 0.33	0.088	1.279	0.294
C_11_H_11_N_3_O_4_ (ng/m^3^)	84	0.30 ± 0.29	0.132	1.334	0.247
C_15_H_10_N_2_O_4_ (ng/m^3^)	84	0.28 ± 0.99	0.054	6.357	0.014
C_15_H_13_N (ng/m^3^)	84	0.27 ± 0.41	0.043	1.736	0.302
C_17_H_15_NO_3_ (ng/m^3^)	84	0.27 ± 0.20	0.156	1.077	0.207
C_10_H_7_F_2_N_3_ (ng/m^3^)	84	0.30 ± 0.33	0.088	1.279	0.294
C_13_H_17_N_3_O_4_ (ng/m^3^)	84	0.23 ± 0.30	0.110	2.166	0.028
C_12_H_16_N_4_O_2_S (ng/m^3^)	84	0.22 ± 0.35	0.050	2.076	0.232
C_16_H_15_N_3_O_2_ (ng/m^3^)	84	0.22 ± 0.23	0.075	0.929	0.239
C_12_H_10_O_2_S (ng/m^3^)	84	0.21 ± 0.93	0.040	6.106	0.062
Esters (Est)					
C_12_H_22_O_7_ (ng/m^3^)	84	2.40 ± 10.80	0.231	71.042	0.969
C_3_H_6_O_3_ (ng/m^3^)	84	1.53 ± 6.92	0.296	45.540	0.332
C_9_H_18_Cl_3_O_4_P (ng/m^3^)	84	0.84 ± 3.55	0.152	23.396	0.294
C_24_H_20_F_10_O_4_ (ng/m^3^)	84	0.74 ± 3.32	0.064	21.848	0.416
C_20_H_24_O_2_ (ng/m^3^)	84	0.57 ± 2.76	0.051	18.099	0.252
C_28_H_46_O_2_ (ng/m^3^)	84	0.46 ± 0.54	0.040	2.683	0.753
C_14_H_24_O_3_Si_2_ (ng/m^3^)	84	0.38 ± 1.61	0.088	10.628	0.088
C_15_H_15_F_5_O_4_ (ng/m^3^)	84	0.37 ± 1.45	0.059	9.584	0.186
C_16_H_20_O_6_ (ng/m^3^)	84	0.31 ± 0.52	0.051	2.174	0.154
C_13_H_18_O_3_ (ng/m^3^)	84	0.21 ± 0.28	0.024	0.952	0.243
C_28_H_30_O_4_ (ng/m^3^)	84	0.20 ± 0.24	0.037	1.022	0.299
Alcohols (Alc)					
C_8_H_18_O (ng/m^3^)	84	2.33 ± 10.66	0.283	70.066	0.857
Amines (Am)					
C_13_H_22_N_2_O (ng/m^3^)	84	0.45 ± 0.99	0.105	6.405	0.405
C_19_H_23_NO (ng/m^3^)	84	0.19 ± 0.31	0.056	1.407	0.056
Ketones (Ket)					
C_15_H_26_O (ng/m^3^)	84	0.44 ± 0.51	0.082	2.372	0.676
C_16_H_14_O (ng/m^3^)	84	0.24 ± 0.34	0.077	1.867	0.146

**Table 3:** Multi-factor generalized linear regression analysis of indoor PM_2.5_ exposure.

Variable	Estimate	SE	Stat	*p*
Gender (male vs. female)	0.720	1.074	0.670	0.505
Age	3.816	1.128	3.384	**0.001**
Outdoor PM_2.5_	0.642	0.637	1.007	0.317
T	0.141	0.214	0.662	0.510
Rhum	−0.085	0.058	−1.460	0.149
Education	1.548	1.162	1.332	0.188
Resident years (≤10 vs. >10)	−4.593	3.881	−1.183	0.241
Area (≤120 vs. >120)	5.339	1.673	3.190	**0.002**
Indoor time (<20 vs. ≥20)	−2.045	1.814	−1.128	0.264
Smoking (Yes vs. No)	−0.660	2.027	−0.326	0.746
Passive smoking (Yes vs. No)	−2.442	1.405	−1.738	0.087
Large furniture (Yes vs. No)	−0.199	1.449	−0.137	0.891
Floor material	1.053	0.851	1.238	0.220
Sofa use time (≤8 vs. >8)	−2.077	2.060	−1.008	0.317
Air chemical (Yes vs. No)	−0.419	1.439	−0.291	0.772
Air humidifier (Yes vs. No)	−6.759	3.013	−2.244	**0.028**
Dry cleaning times (≤3 vs. >3)	0.667	2.174	0.307	0.760
Indoor burning incense (Yes vs. No)	−0.013	1.592	−0.008	0.994
Exercises per day	1.040	0.632	1.647	0.104

Note: The bolded *p*-values in the table indicate statistical significance (*p* < 0.05).

**Table 4:** Basic characteristics of chemical components in individual PM_2.5_.

	N	Mean ± SD	Minimum	Maximum	IQR
T (°C)	258	29.57 ± 2.28	15.370	34.500	2.170
Rhum (%)	258	74.76 ± 12.03	31.681	99.000	16.291
Individual PM_2.5_ (μg/m^3^)	258	38.06 ± 48.38	2.684	268.947	27.754
Esters (Est)					
C_28_H_46_O_2_ (ng/m^3^)	258	50.37 ± 444.62	1.827	7118.377	25.027
C_13_H_18_O_3_ (ng/m^3^)	258	42.76 ± 396.85	0.712	6353.121	20.881
C_17_H_16_O_3_Si (ng/m^3^)	258	30.81 ± 252.64	1.341	3952.810	9.036
C_28_H_30_O_4_ (ng/m^3^)	258	18.73 ± 73.86	0.809	1085.231	13.633
C_15_H_15_F_5_O_4_ (ng/m^3^)	258	16.16 ± 123.64	0.611	1946.794	6.658
C_24_H_20_F_10_O_4_ (ng/m^3^)	258	15.37 ± 157.83	0.192	2534.171	6.245
C_14_H_24_O_3_Si_2_ (ng/m^3^)	258	14.35 ± 54.89	0.702	642.766	11.082
C_14_H_22_O_2_ (ng/m^3^)	258	12.02 ± 31.85	1.861	415.086	7.779
C_17_H_13_ClO_4_ (ng/m^3^)	258	8.88 ± 54.67	0.546	685.089	2.992
C_20_H_24_O_2_ (ng/m^3^)	258	6.88 ± 44.57	0.726	701.380	1.955
C_17_H_19_F_5_O_4_ (ng/m^3^)	258	6.64 ± 17.54	0.626	150.191	5.579
Aromatic hydrocarbon derivatives (ArD)					
C_17_H_15_NO_3_ (ng/m^3^)	258	27.28 ± 243.46	1.071	3892.709	11.227
C_13_H_13_F_5_O_2_ (ng/m^3^)	258	21.91 ± 114.7	0.973	1783.835	16.131
C_15_H_11_N_3_O_3_ (ng/m^3^)	258	19.18 ± 143.71	0.178	2283.447	10.820
C_11_H_11_BrO_4_ (ng/m^3^)	258	18.23 ± 88.32	0.830	1159.188	13.094
C_10_H_7_F_2_N_3_ (ng/m^3^)	258	17.19 ± 31.65	1.095	262.154	21.675
C_16_H_9_Cl (ng/m^3^)	258	16.15 ± 53.44	0.134	534.897	1.510
C_11_H_11_N_3_O_4_ (ng/m^3^)	258	12.05 ± 31.42	1.441	277.919	9.875
C_12_H_9_F_2_NO_3_ (ng/m^3^)	258	10.39 ± 25.05	1.663	217.695	2.532
C_21_H_30_O_3_Si (ng/m^3^)	258	8.89 ± 26.95	0.229	270.199	6.202
C_11_H_10_FNS (ng/m^3^)	258	7.35 ± 18.92	0.084	152.050	5.667
C_35_H_20_N_4_O_12_ (ng/m^3^)	258	6.42 ± 17.77	0.212	184.260	5.874
C_14_H_11_N_3_S (ng/m^3^)	258	4.50 ± 28.82	0.212	392.345	0.372
C_14_H_9_Cl (ng/m^3^)	258	4.32 ± 3.87	0.040	34.641	0.287
Amines (Am)					
C_11_H_13_NO_3_ (ng/m^3^)	258	23.6 ± 152.83	0.914	2418.421	16.477
C_12_H_16_N_2_O_3_ (ng/m^3^)	258	23.55 ± 55.01	0.734	561.070	21.851
C_8_H_13_N_7_ (ng/m^3^)	258	20.36 ± 39.82	0.827	317.893	21.104
C_18_H_19_NO_2_ (ng/m^3^)	258	17.96 ± 192.25	0.700	3082.024	1.656
C_13_H_17_N_3_O_4_ (ng/m^3^)	258	15.46 ± 35.91	0.502	344.048	17.968
C_13_H_22_N_2_O (ng/m^3^)	258	14.99 ± 60.17	1.078	843.424	7.405
C_13_H_23_F_3_N_2_O_2_ (ng/m^3^)	258	14.02 ± 37.5	0.052	370.767	12.770
C_14_H_20_N_2_O_4_S (ng/m^3^)	258	12.92 ± 48.75	0.357	641.283	10.456
Nitrogen-containing organic compounds (NOCs)					
C_15_H_13_N (ng/m^3^)	258	26.44 ± 152.2	1.321	2317.884	13.240
C_6_H_5_N_7_O_2_ (ng/m^3^)	258	19.39 ± 81.01	0.496	1230.048	14.683
C_12_H_17_N_4_OPS_2_ (ng/m^3^)	258	14.06 ± 93.88	0.608	1499.131	9.956
C_16_H_15_N_3_O_2_ (ng/m^3^)	258	9.65 ± 18.32	0.589	142.836	10.639
Silicon-containing organic compounds (SOCs)					
C_14_H_24_OSi (ng/m^3^)	258	27.66 ± 149.63	1.063	2357.927	21.206
C_15_H_26_N_4_O_3_Si (ng/m^3^)	258	11.95 ± 34.64	0.589	477.737	12.678
C_25_H_50_OSi (ng/m^3^)	258	8.8 ± 29.92	0.746	364.382	6.513
C_22_H_30_OSi (ng/m^3^)	258	6.35 ± 19.13	0.509	223.605	3.370

**Table 5:** Multi-factor generalized linear regression analysis of individual PM_2.5_ exposure.

Variable	Estimate	SE	Stat	*p*
Gender (male vs. female)	0.099	0.196	0.505	0.614
Age (≤70 vs. >70)	0.014	0.170	0.085	0.933
T	−0.003	0.035	−0.082	0.935
Rhum	0.000	0.007	0.063	0.950
Smoking (Yes vs. No)	−0.244	0.299	−0.815	0.416
Cooking (Yes vs. No)	0.025	0.192	0.132	0.895
Air conditioners (≤2 vs. >2)	−0.317	0.162	−1.957	0.051
Air chemical (Yes vs. No)	0.091	0.163	0.557	0.578
Passive smoking per day	−0.365	0.173	−2.112	**0.036**
Drinking (Yes vs. No)	0.015	0.221	0.066	0.948

Note: The bolded *p*-values in the table indicate statistical significance (*p* < 0.05).

## Data Availability

The data presented in this study are available on request from the corresponding author. The data are not publicly available due to restrictions privacy.

## Supplementary Materials

The following supporting information can be downloaded at: https://www.mdpi.com/article/10.3390/toxics14050414/s1, Table S1: Basic characteristics of chemical components in indoor PM_2.5_ (ng/m^3^); Table S2: Basic characteristics of chemical components in individual PM_2.5_ (ng/m^3^); Table S3: Wilcoxon signed-rank test comparing individual and indoor PM_2.5_ concentrations; Table S4: T-test of the difference (individual–outdoor) in PM_2.5_ concentrations. Figure S1: Indoor PM_2.5_ concentrations across study participants; Figure S2: Individual PM_2.5_ exposure concentrations across study participants.

## Appendix A

**Table A1:** Single-factor analysis results of indoor PM_2.5_ exposure.

	Mean ± SD/N (%)	*R*	*p*
Outdoor PM_2.5_	11.62 ± 1.73	0.441	**<0.001**
Individual PM_2.5_	42.11 ± 40.43	−0.115	0.298
T	29.26 ± 2.87	−0.272	**0.012**
Rhum	73.23 ± 11.35	0.298	**0.006**
Age		0.036	0.745
≤70	50 (59.52)		
>70	34 (40.48)		
Gender		−0.032	0.775
Female	46 (54.76)		
Male	38 (45.24)		
BMI		0.012	0.916
<18.5	4 (4.76)		
18.5–24	48 (57.14)		
≥24	32 (38.10)		
Education		0.378	**<0.001**
Below Senior Secondary Education	58 (69.05)		
Senior Secondary Education or Above	26 (30.95)		
Residence years		−0.402	**<0.001**
≤10	8 (9.52)		
>10	76 (90.48)		
Area		0.400	**<0.001**
≤120 m^2^	60 (71.43)		
>120 m^2^	24 (28.57)		
Floor		−0.120	0.275
≤3	17 (15.2)		
>3	21 (18.8)		
Close to major traffic arteries		0.166	0.131
No	14 (16.67)		
Yes	70 (83.33)		
Smoking		−0.326	**0.002**
No	66 (78.57)		
Yes	18 (21.43)		
Drinking		−0.130	0.238
No	68 (80.95)		
Yes	16 (19.05)		
Passive smoking		−0.219	**0.045**
No	62 (73.81)		
Yes	22 (26.19)		
Exercises per day		0.226	**0.039**
Outdoor PM_2.5_	11.62 ± 1.73	0.441	**<0.001**
Individual PM_2.5_	42.11 ± 40.43	−0.115	0.298
<0.5 h	20 (23.81)		
0.5–1 h	26 (30.95)		
>1 h	38 (45.24)		
Indoor time		0.222	**0.042**
<20 h	8 (9.52)		
≥20 h	76 (90.48)		
Cooking		0.022	0.844
No	60 (71.43)		
Yes	24 (28.57)		
Cooking fuel		−0.191	0.082
Coal gas	54 (64.29)		
Other	30 (35.71)		
Whether air purifier is used?		−0.172	0.118
No	78 (92.86)		
Yes	6 (7.14)		
Whether air humidifier is used?		−0.258	**0.018**
No	80 (95.24)		
Yes	4 (4.76)		
Whether air chemical is used?		−0.254	**0.020**
No	42 (50.00)		
Yes	42 (50.00)		
Dry cleaning times per week		0.246	**0.024**
≤3	10 (11.90)		
>3	74 (88.10)		
Summer ventilation frequency		−0.018	0.868
No	4 (4.76)		
Yes	80 (95.24)		
Indoor burning incense		0.409	**<0.001**
No	36 (42.86)		
Yes	48 (57.14)		
Large furniture buy recently?		−0.300	**0.006**
No	68 (80.95)		
Yes	16 (19.05)		
Air conditioning per day		−0.192	0.081
≤8 h	46 (54.76)		
> 8 h	38 (45.24)		
Sofa use time		0.231	**0.035**
≤8 h	74 (88.10)		
>8 h	10 (11.90)		
Floor material		0.334	**0.002**
Ceramic tile	40 (47.62)		
Wood	18 (21.43)		
Other	26 (30.95)		

Note: The bolded *p*-values in the table indicate statistical significance (*p* < 0.05).

**Table A2:** Single-factor analysis results of Individual PM_2.5_ exposure.

	Mean ± SD/N (%)	*R*	*p*
Outdoor PM_2.5_	12.33 ± 3.97	0.267	**<0.001**
Indoor PM_2.5_	8.99 ± 6.54	−0.009	0.886
T	29.57 ± 2.28	−0.031	0.623
Rhum	74.76 ± 12.03	−0.020	0.745
Age		−0.014	0.821
≤70	114 (44.19)		
> 70	144 (55.81)		
Gender		0.049	0.436
Female	46 (54.76)		
Male	38 (45.24)		
BMI		0.045	0.467
<18.5	8 (3.10)		
18.5–24	126 (48.84)		
≥24	124 (48.06)		
Educational		0.022	0.719
Below Senior Secondary Education	142 (55.04)		
Senior Secondary Education or above	116 (44.96)		
Residence years (Year)		−0.064	0.305
≤10	72 (27.91)		
>10	186 (72.09)		
Area		−0.050	0.419
≤120 m^2^	138 (53.49%)		
>120 m^2^	120 (46.51%)		
Floor		−0.009	0.890
≤3	128 (49.61)		
>3	130 (50.39)		
Close to major traffic arteries		0.103	0.099
No	54 (20.93)		
Yes	204 (79.07)		
Smoking		0.092	0.279
No	227 (87.98)		
Yes	31 (12.02)		
Drinking		−0.029	0.641
No	214 (82.95)		
Yes	44 (17.05)		
Passive smoking per day		−0.139	**0.025**
<0.5 h	226 (87.60)		
0.5–1 h	16 (6.20)		
>1 h	16 (6.20)		
Exercises per day		−0.050	0.426
<0.5 h	44 (17.05)		
0.5–1 h	91 (35.27)		
>1 h	123 (47.67)		
Indoor time		0.051	0.415
<20 h	41 (15.89)		
≥20 h	217 (84.11)		
Cooking		−0.068	0.273
No	81 (31.40)		
Yes	177 (68.60)		
Cooking fuel		−0.029	0.643
Coal gas	62 (75.97)		
Other	196 (24.03)		
Whether air purifier is used?		−0.030	0.632
No	230 (89.15)		
Yes	28 (10.85)		
Whether air humidifier is used?		−0.005	0.936
No	244 (94.57)		
Yes	14 (5.43)		
Whether air chemical is used?		0.128	**0.041**
No	138 (53.49)		
Yes	120 (46.51)		
Dry cleaning times per week		−0.104	0.095
≤3	48 (18.60)		
>3	210 (81.40)		
Summer ventilation frequency		0.017	0.783
No	28 (10.85)		
Yes	230 (89.15)		
Indoor burning incense		−0.027	0.668
No	140 (54.26)		
Yes	118 (45.74)		
Large furniture buy recently?		0.017	0.785
No	216 (83.72)		
Yes	42 (16.28)		
Number of air conditioners		−0.123	**0.048**
≤2	146 (56.59)		
>2	112 (43.41)		
Sofa use time		0.058	0.357
≤8 h	241 (93.41)		
>8 h	17 (6.59)		
			−0.020
Ceramic tile		145 (56.20)	
Wood		61 (23.64)	
Other		52 (20.16)	

Note: Bolded *p* values in the table indicate statistical significance (*p* < 0.05).

**Table A3:** Uni-variate regression analysis of common chemical component exposure (Individual vs. Indoor).

Variable	Estimate	SE	Stat	FDR_*p*
Amines (Am)				
C_16_H_18_N_2_O_2_	0.008	0.109	0.070	0.975
C_17_H_19_N_3_O	0.080	0.728	0.110	0.913
C_16_H_28_N_2_O_2_S	0.060	0.526	0.114	0.910
C_9_H_18_N_2_	−166.664	94.527	−1.763	0.383
C_10_H_12_N_4_O_2_S_2_	0.266	6.536	0.041	0.999
C_15_H_22_F_7_NO	0.583	0.914	0.638	0.936
C_18_H_17_N_3_O_3_S	46.688	9.898	4.717	**0.001**
C_10_H_17_NO_3_	0.038	0.259	0.145	0.914
C_19_H_22_N_2_O_2_S	6.335	30.419	0.208	0.979
Aromatic hydrocarbon derivatives (ArD)				
C_14_H_10_FN_3_O	4.760	5.085	0.936	1.000
C_15_H_11_NO_4_	1.234	5.168	0.239	0.975
C_19_H_25_NO_3_	0.470	3.999	0.118	0.995
C_11_H_5_N_3_O_2_	0.017	0.386	0.045	0.993
C_6_H_4_N_4_O_3_	−0.058	1.927	−0.030	0.989
C_9_H_11_NO	−0.034	0.455	−0.076	0.982
C_10_H_10_FN_3_	−0.617	1.543	−0.400	0.999
Nitro-containing organic compounds (NOCs)				
C_10_H_17_N_5_	0.152	0.067	2.265	0.443
C_5_H_9_NO_2_	2.533	51.186	0.049	0.961
C_7_H_4_N_4_	−0.151	0.214	−0.706	1.000
C_17_H_24_N_4_O_4_S	−0.154	0.558	−0.276	0.921
C_20_H_35_N_3_O_2_	−0.001	0.009	−0.098	0.948
C_5_H_3_ClIN	−0.091	0.228	−0.400	0.999
Hydrocarbons (Hyd)				
C_3_H_3_Br	−60.860	22.510	−2.704	0.059
C_2_H_3_F_3_	−71.704	43.779	−1.638	0.801
CH_2_BrNO_2_	−0.002	0.004	−0.544	0.995
C_3_Cl_2_F_6_	−0.003	0.053	−0.065	0.984
C_6_H_14_S	0.074	0.487	0.153	0.941
Ketones (Ket)				
C_6_H_4_O	1.818	3.949	0.460	0.982
C_24_H_26_N_2_O	−0.988	2.897	−0.341	0.870
C_7_H_7_F_5_O_2_	0.021	0.171	0.122	0.965
Esters (Est)				
C_26_H_26_O_6_	−0.312	0.509	−0.613	0.910
C_13_H_16_O_3_	−8.310	26.038	−0.319	0.950
C_21_H_16_O_5_	2.621	0.367	7.144	**<0.001**
C_14_H_9_F_8_NO_6_	−1.084	6.718	−0.161	0.985
C_8_H_16_N_2_O_2_	132.126	130.371	1.013	0.997
C_5_H_11_NOS	0.373	5.276	0.071	0.997
C_18_H_21_F_5_O_4_	0.338	2.795	0.121	0.923
C_2_H_5_O_3_P	−0.098	5.767	−0.017	0.987
C_14_H_17_FO_4_	0.010	0.055	0.176	0.943
C_17_H_15_ClO_4_	−0.007	0.072	−0.098	0.948

Note: The bolded *p*-values in the table indicate statistical significance (*p* < 0.05).
