# Supplementary material for: From Environmental Concentrations to Individual Inhalation: Analysis of Exposure Differences to PM2.5 and Chemical Components in Elderly Populations and Their Influencing Factors
Source: Toxics. 2026 May 10;14(5):414. doi: 10.3390/toxics14050414 (PMC13211666; doi:10.3390/toxics14050414)
Supplement: Supplementary file 1 [file toxics-14-00414-s001.zip › Supplementary Tables.pdf]

Supplementary Table

Table S1. Basic characteristics of chemical components in indoor $PM_{2.5}$ (ng/m^3)

Components	CAS Number	Mean ± SD	Minimum	Maximum	LOD (ng/m^3)
$C_4H_{11}FSi$	10132-71-5	0.19 ± 0.14	0.153	0.768	0.009
$C_9H_{12}O_2$	6180-61-6	0.19 ± 0.10	0.152	0.468	0.012
$C_{10}H_{12}N_2O$	13732-32-6	0.19 ± 0.10	0.152	0.483	0.013
$C_{14}H_{35}O_3PSi_2$	1000273-30-1	0.19 ± 0.08	0.163	0.517	0.025
$C_{13}H_{14}O_5$	52918-79-3	0.19 ± 0.06	0.175	0.423	0.019
$C_4H_4I_2N_2$	37067-96-2	0.19 ± 0.05	0.178	0.357	0.025
$C_9H_{10}O$	22927-13-5	0.19 ± 0.04	0.183	0.366	0.010
$C_{20}H_{28}N_2$	82605-37-6	0.19 ± 0.04	0.180	0.360	0.022
C_6H_4O	31097-80-0	0.19 ± 0.04	0.181	0.362	0.008
$C_{11}H_{12}O_4$	93198-71-1	0.19 ± 0.04	0.182	0.365	0.016
$C_{10}H_{12}N_2O_3$	1000240-23-5	0.19 ± 0.04	0.182	0.364	0.016
$C_{14}H_{13}F_5O_4$	1000415-77-4	0.19 ± 0.04	0.181	0.362	0.025
$C_{22}H_{36}O_4Si_3$	1000347-47-5	0.19 ± 0.04	0.183	0.365	0.033
$C_2H_3N_3$	288-36-8	0.19 ± 0.04	0.183	0.365	0.006
$C_{20}H_{43}O_3P$	1000323-27-0	0.19 ± 0.04	0.183	0.366	0.027
C_8ClF_9	136264-68-1	0.19 ± 0.04	0.183	0.366	0.022
$C_{15}H_{26}O_3Si_2$	55724-93-1	0.19 ± 0.04	0.178	0.355	0.023
$C_{16}H_{28}O_4$	1000339-14-8	0.19 ± 0.04	0.178	0.357	0.021
$C_{14}H_{15}ClF_4O_2$	1000343-78-5	0.19 ± 0.04	0.186	0.372	0.024
$C_{21}H_{34}N_2O_2$	1000402-50-4	0.19 ± 0.04	0.177	0.355	0.025
$C_{15}H_{12}Cl_2O_2$	1000331-31-5	0.19 ± 0.03	0.190	0.379	0.022
$C_{10}H_{18}O$	10482-56-1	0.19 ± 0.03	0.186	0.371	0.012
$C_{18}H_{13}N_3O_2S_2$	1000303-07-4	0.19 ± 0.03	0.183	0.365	0.027
$C_{15}H_{18}F_4N_2O_3$	1000224-16-1	0.19 ± 0.03	0.188	0.375	0.026
$C_{15}H_{12}N_2O_5$	350795-29-8	0.18 ± 0.22	0.082	0.857	0.022
$C_{35}H_{20}N_4O_{12}$	301209-92-7	0.18 ± 0.19	0.029	0.665	0.050
$C_{19}H_{19}NO_5$	1000276-12-4	0.18 ± 0.06	0.160	0.409	0.025
$C_{14}H_{29}O_4PS$	129065-11-8	0.18 ± 0.05	0.163	0.334	0.024
$C_{10}H_{14}$	933-98-2	0.18 ± 0.04	0.168	0.336	0.011
$C_{18}H_{25}NO_3$	1000323-91-7	0.18 ± 0.04	0.164	0.337	0.022
$C_{13}H_9ClO_2$	38569	0.18 ± 0.04	0.173	0.345	0.017
$C_{20}H_{24}O_4Si$	1000363-25-7	0.18 ± 0.04	0.168	0.335	0.026
$C_6H_{15}N$	142-84-7	0.18 ± 0.04	0.171	0.341	0.008
C_9H_9NO	24966-13-0	0.18 ± 0.04	0.173	0.346	0.011
$C_{12}H_{15}N_3O_3S$	331758-59-9	0.18 ± 0.04	0.175	0.350	0.021
C_2H_7NS	60-23-1	0.18 ± 0.04	0.169	0.338	0.006

$C_{16}H_{17}F_5O_4$	1000392-10-6	0.18 ± 0.04	0.174	0.349	0.027

Table S1 (cont.). Basic characteristics of chemical components in indoor $PM_{2.5}$ (ng/m³)

Components	CAS Number	Mean ± SD	Minimum	Maximum	LOD （ng/m³）
$C_{25}H_{22}O_5$	92206-04-7	0.18 ± 0.03	0.174	0.349	0.029
$C_{13}H_{12}ClNO_2S$	17969-12-9	0.18 ± 0.03	0.173	0.347	0.021
$C_8H_4Cl_2N_4O$	1000350-96-2	0.18 ± 0.03	0.181	0.361	0.018
$C_{13}H_{12}N_2O$	1000144-83-9	0.18 ± 0.03	0.177	0.354	0.016
$C_5H_{11}NOS$	692-99-9	0.18 ± 0.03	0.179	0.358	0.010
$C_{23}H_{25}NO_6$	1000287-15-2	0.17 ± 0.27	0.076	1.223	0.030
$C_{15}H_{26}O_2$	55823-65-9	0.17 ± 0.15	0.120	0.821	0.018
$C_{14}H_{22}N_2O_2$	1000401-36-9	0.17 ± 0.11	0.149	0.867	0.019
$C_{11}H_7F_6NO_2$	55734-40-2	0.17 ± 0.10	0.146	0.575	0.022
$C_9H_6O_6$	554-95-0	0.17 ± 0.09	0.140	0.551	0.016
$C_{22}H_{16}O$	6639-05-0	0.17 ± 0.09	0.134	0.491	0.022
$C_{14}H_{20}N_2O_4S$	1334427-13-2	0.17 ± 0.08	0.143	0.446	0.023
$C_{14}H_{12}N_2$	2622-63-1	0.17 ± 0.08	0.137	0.406	0.016
$C_{22}H_{17}NO_2$	1000304-84-1	0.17 ± 0.07	0.153	0.426	0.024
$C_7H_8N_2O$	114-33-0	0.17 ± 0.05	0.148	0.309	0.011
$C_{15}H_{26}N_2O$	1000194-74-4	0.17 ± 0.05	0.156	0.364	0.019
$C_{13}H_{18}O_2$	5406-57-5	0.17 ± 0.04	0.163	0.326	0.016
$C_{17}H_{18}N_4O_4$	1000410-68-0	0.17 ± 0.04	0.164	0.329	0.025
$C_9H_8O_2$	579-07-7	0.17 ± 0.04	0.165	0.330	0.011
$C_7H_{16}O$	2313-61-3	0.17 ± 0.04	0.164	0.329	0.009
$C_3H_5NO_2$	17082-05-2	0.17 ± 0.04	0.167	0.334	0.007
$C_6H_6N_2$	26187-27-9	0.17 ± 0.04	0.165	0.331	0.009
$C_{16}H_{26}N_2O_2$	94-15-5	0.17 ± 0.04	0.163	0.326	0.021
$C_{10}H_{22}O_4$	143-22-6	0.17 ± 0.04	0.166	0.331	0.016
$C_{11}H_{12}O$	827-69-0	0.17 ± 0.04	0.164	0.328	0.012
$C_4H_5N_3O$	6863-77-0	0.17 ± 0.04	0.164	0.328	0.009
$C_{11}H_{17}NO_2$	1000156-48-0	0.17 ± 0.04	0.166	0.332	0.015
$C_{15}H_{29}NO$	1000458-92-1	0.17 ± 0.04	0.159	0.318	0.018
$C_{18}H_{19}NO_2$	24695-70-3	0.17 ± 0.04	0.163	0.327	0.021
$C_{17}H_{15}FO_4$	1000357-53-7	0.17 ± 0.04	0.165	0.330	0.022
$C_6H_{10}O_2$	6971-63-7	0.17 ± 0.03	0.160	0.320	0.009
C_6H_7N	1516-01-4	0.17 ± 0.03	0.159	0.319	0.008
$C_7H_{18}N_2Si_2$	1000-70-0	0.17 ± 0.03	0.158	0.316	0.014
$C_9H_{20}O_2$	1000334-81-5	0.17 ± 0.03	0.170	0.340	0.012
$C_{18}H_{19}NO_2$	5144-20-7	0.17 ± 0.03	0.161	0.322	0.021
$C_5H_{10}O_2$	694-54-2	0.17 ± 0.03	0.167	0.334	0.008
$C_8H_9N_3$	55463-64-4	0.17 ± 0.03	0.158	0.316	0.011

$C_{12}H_{20}N_2OS$	283170-57-0	0.17 ± 0.03	0.158	0.316	0.018
$C_{16}H_{34}O_3S$	1000309-17-9	0.17 ± 0.03	0.162	0.324	0.023
$C_{19}H_{19}BrO_2$	1000370-38-6	0.16 ± 0.24	0.079	1.279	0.026

Table S1 (cont.). Basic characteristics of chemical components in indoor $PM_{2.5}$ (ng/m^3)

Components	CAS Number	Mean ± SD	Minimum	Maximum	LOD （ng/m³）
$C_{23}H_{28}O_6$	1000358-58-3	0.16 ± 0.21	0.059	0.841	0.029
$C_{20}H_{14}N_2$	1684-14-6	0.16 ± 0.20	0.102	0.988	0.021
C_6H_5BrClN	21402-26-6	0.16 ± 0.20	0.085	0.949	0.016
$C_9H_9N_3O_3$	1000267-28-6	0.16 ± 0.09	0.112	0.354	0.016
$C_{17}H_{15}NO_6$	760195-09-3	0.16 ± 0.07	0.130	0.391	0.024
$C_{10}H_{12}O_3$	28129-15-9	0.16 ± 0.05	0.148	0.305	0.014
$C_9H_9N_3O_2$	24410-25-1	0.16 ± 0.05	0.136	0.277	0.015
$C_6H_{15}O_4P$	78-40-0	0.16 ± 0.04	0.145	0.289	0.014
$C_{10}H_{10}O_2$	1000188-08-0	0.16 ± 0.04	0.149	0.299	0.012
$C_{12}H_{16}O$	1131-60-8	0.16 ± 0.03	0.148	0.296	0.013
$C_{15}H_{20}O_2$	1000293-32-1	0.16 ± 0.03	0.156	0.312	0.017
$C_{16}H_{34}S_2$	29956-99-8	0.16 ± 0.03	0.149	0.299	0.022
$C_7H_{16}FO_2P$	660-21-9	0.16 ± 0.03	0.149	0.299	0.014
$C_4H_{11}NO$	1000322-43-1	0.16 ± 0.03	0.157	0.314	0.007
$C_9H_{10}O_3$	1000282-75-9	0.16 ± 0.03	0.157	0.313	0.013
$C_{21}H_{26}N_2O_3$	5552-25-0	0.16 ± 0.03	0.152	0.304	0.026
$C_{11}H_{23}Br$	127839-47-8	0.16 ± 0.03	0.156	0.313	0.018
$C_{13}H_{24}OSi$	65115-51-7	0.16 ± 0.03	0.151	0.302	0.017
$C_{10}H_8N_2$	553-26-4	0.16 ± 0.03	0.152	0.305	0.012
$C_{14}H_{25}NO_4$	1000325-15-4	0.16 ± 0.03	0.149	0.298	0.020
$C_{10}H_8F_3NO_3$	1000299-62-8	0.16 ± 0.03	0.152	0.303	0.018
$C_{11}H_{15}NO_2Si$	21654-63-7	0.16 ± 0.03	0.156	0.312	0.017
$C_{12}H_{10}N_2$	1000303-41-6	0.16 ± 0.03	0.155	0.309	0.014
$C_{17}H_{18}O_3$	34883-17-5	0.16 ± 0.03	0.153	0.307	0.020
$C_8H_5N_3O_2S_2$	40045-73-6	0.16 ± 0.03	0.149	0.298	0.018
$C_{12}H_{19}N_5O_3$	1000304-55-6	0.16 ± 0.03	0.155	0.311	0.021
$C_{14}H_{10}N_4OS$	1000105-31-2	0.16 ± 0.03	0.152	0.305	0.021
$C_8H_{16}O$	15726-15-5	0.16 ± 0.03	0.148	0.296	0.010
$C_{17}H_{19}N_3O$	1000302-50-5	0.16 ± 0.02	0.152	0.305	0.021
$C_{13}H_{14}O_6$	1000158-50-7	0.16 ± 0.02	0.157	0.315	0.020
$C_{16}H_{15}N_3S$	1000242-18-7	0.15 ± 0.23	0.060	1.033	0.021
$C_{18}H_{14}N_6O_2$	1000459-95-3	0.15 ± 0.17	0.034	0.584	0.025
$C_{11}H_{13}N_5O_2S$	27042-75-7	0.15 ± 0.13	0.093	0.585	0.021
$C_{11}H_6ClNO_4$	1000254-68-4	0.15 ± 0.09	0.120	0.463	0.019
$C_{19}H_{37}NO_4$	1000392-67-4	0.15 ± 0.08	0.115	0.364	0.025
$C_{12}H_{17}N_4OPS_2$	1000337-35-6	0.15 ± 0.08	0.101	0.312	0.024
$C_{15}H_{15}N_5O$	1000296-81-0	0.15 ± 0.07	0.127	0.412	0.021
$C_6H_5N_3O_2$	65996-50-1	0.15 ± 0.06	0.130	0.397	0.012

$C_{12}H_{12}V$	12636-68-9	0.15 ± 0.06	0.133	0.398	0.016

Table S1 (cont.). Basic characteristics of chemical components in indoor $PM_{2.5}$ (ng/m^3)

Components	CAS Number	Mean ± SD	Minimum	Maximum	LOD (ng/m³)
$C_{17}H_{15}FO_5$	1000390-98-2	0.15 ± 0.06	0.137	0.390	0.023
$C_{14}H_{22}N_2O_2$	1000190-75-5	0.15 ± 0.05	0.128	0.266	0.019
$C_{20}H_{26}OSi$	138983-00-3	0.15 ± 0.04	0.136	0.279	0.023
$C_{18}H_{20}O$	1000110-34-9	0.15 ± 0.04	0.141	0.284	0.019
$C_6H_{16}NOPS$	1000440-57-6	0.15 ± 0.03	0.138	0.277	0.014
$C_{20}H_{30}N_2O_2$	98262-59-0	0.15 ± 0.03	0.144	0.288	0.024
$C_{10}H_{14}O_5V$	3153-26-2	0.15 ± 0.03	0.139	0.278	0.020
$C_{12}H_{11}F_3N_4$	1000264-96-1	0.15 ± 0.03	0.147	0.294	0.020
$C_{17}H_{26}O_4Si$	1000352-41-7	0.15 ± 0.03	0.140	0.280	0.024
$C_9H_{15}ClF_2O_2$	1000376-27-0	0.15 ± 0.03	0.147	0.293	0.017
C_7H_8OS	13679-73-7	0.15 ± 0.03	0.139	0.279	0.011
$C_6H_6N_2O$	1453-82-3	0.15 ± 0.03	0.146	0.293	0.010
$C_9H_{14}O_4$	6439-57-2	0.15 ± 0.03	0.144	0.289	0.014
$C_8H_5N_3O_4$	4771-15-7	0.15 ± 0.03	0.139	0.278	0.016
$C_9H_{14}O_3$	38653-27-9	0.15 ± 0.03	0.139	0.278	0.013
$C_{11}H_9F_5O_2$	1000308-00-2	0.15 ± 0.03	0.141	0.282	0.020
$C_{15}H_{32}O_3S$	1000309-12-3	0.15 ± 0.03	0.141	0.282	0.022
$C_{10}H_8F_5NO$	1000307-33-0	0.15 ± 0.03	0.142	0.285	0.019
$C_{10}H_{10}O_3$	1603-79-8	0.15 ± 0.02	0.146	0.292	0.014
$C_{10}H_{18}O$	98-55-5	0.15 ± 0.02	0.147	0.294	0.012
$C_{18}H_{18}O_4$	1000325-06-7	0.15 ± 0.02	0.149	0.298	0.022
$C_{10}H_{16}O$	4884-24-6	0.15 ± 0.02	0.149	0.298	0.012
$C_{15}H_{16}N_4O_4$	324012-36-4	0.15 ± 0.02	0.142	0.284	0.023
$C_8H_5F_4NO$	1000307-30-8	0.14 ± 0.23	0.064	0.989	0.016
$C_8H_9N_5O_2$	1000316-75-8	0.14 ± 0.19	0.067	0.821	0.016
$C_{10}H_9NO_4$	1000396-10-6	0.14 ± 0.18	0.083	0.683	0.016
$C_{18}H_{16}O_5$	1000443-99-9	0.14 ± 0.13	0.070	0.553	0.023
$C_{14}H_{16}ClNO$	97994-57-5	0.14 ± 0.12	0.103	0.595	0.019
$C_{17}H_{13}ClO_4$	1000215-35-2	0.14 ± 0.10	0.110	0.648	0.023
$C_{46}H_{60}O_4$	69505-95-9	0.14 ± 0.08	0.114	0.451	0.049
$C_{15}H_{11}N_3O_3$	1000318-35-0	0.14 ± 0.08	0.092	0.292	0.021
$C_{16}H_{10}N_2O_5$	1000294-28-5	0.14 ± 0.08	0.119	0.454	0.023
$C_{17}H_{15}NO_3$	1000423-07-5	0.14 ± 0.07	0.122	0.426	0.021
$C_{20}H_{23}NO_4$	156785-76-1	0.14 ± 0.05	0.129	0.320	0.025
$C_{23}H_{18}O_2$	1000402-36-9	0.14 ± 0.05	0.125	0.371	0.024
$C_8H_4N_2OS_2$	51974-87-9	0.14 ± 0.04	0.124	0.286	0.016
$C_{17}H_{15}NO$	76462-98-1	0.14 ± 0.04	0.122	0.248	0.019

$C_{26}H_{41}NO_3$	1000361-56-4	0.14 ± 0.03	0.138	0.276	0.030
$C_{10}H_{20}O_3$	1000367-04-0	0.14 ± 0.03	0.134	0.267	0.014
$C_{13}H_{13}F_5O_2$	1000467-36-8	0.14 ± 0.03	0.137	0.275	0.022

Table S1 (cont.). Basic characteristics of chemical components in indoor $PM_{2.5}$ (ng/m^3)

**Components**	**CAS Number**	**Mean ± SD**	**Minimum**	**Maximum**	**LOD （ng/m^3）**
$C_{11}H_{13}NOS$	40991-40-0	0.14 ± 0.03	0.132	0.264	0.016
$C_{22}H_{35}NO_4$	1000361-50-9	0.14 ± 0.03	0.130	0.260	0.028
$C_{18}H_{17}NO_5$	1000316-16-9	0.14 ± 0.03	0.133	0.266	0.024
$C_{17}H_{12}N_2O_2S$	1000268-61-3	0.14 ± 0.03	0.137	0.274	0.023
$C_{12}H_{15}NO_5$	5556-76-3	0.14 ± 0.03	0.135	0.270	0.019
$C_4H_8O_3$	22347-47-3	0.14 ± 0.03	0.132	0.264	0.008
$C_5H_9NO_2$	62400-75-3	0.14 ± 0.03	0.131	0.263	0.009
$C_8H_{10}O_2$	501-94-0	0.14 ± 0.03	0.136	0.272	0.011
$C_8H_6O_2$	553-86-6	0.14 ± 0.03	0.134	0.269	0.010
C_6H_8OS	13678-59-6	0.14 ± 0.03	0.132	0.264	0.010
$C_{17}H_{22}Si_2$	58263-56-2	0.14 ± 0.03	0.138	0.276	0.021
$C_{14}H_{24}OSi$	1000373-05-7	0.14 ± 0.03	0.138	0.276	0.018
$C_{15}H_{11}NO_4$	77631-37-9	0.14 ± 0.03	0.134	0.268	0.020
$C_9H_{18}N_2O_2$	1000375-63-0	0.14 ± 0.03	0.135	0.270	0.014
$C_6H_8N_2$	13925-00-3	0.14 ± 0.03	0.137	0.273	0.009
$C_{20}H_{42}$	504-44-9	0.14 ± 0.03	0.137	0.275	0.021
$C_{24}H_{41}NO_4$	1000383-22-2	0.14 ± 0.03	0.133	0.266	0.030
$C_{15}H_{32}$	25117-24-2	0.14 ± 0.03	0.136	0.271	0.016
$C_{15}H_9ClF_4O_2$	1000343-78-7	0.14 ± 0.03	0.134	0.269	0.024
$C_{16}H_{15}N_3O_2$	1000319-51-8	0.14 ± 0.03	0.136	0.272	0.021
$C_{22}H_{18}N_2O_2$	1000241-02-9	0.14 ± 0.03	0.133	0.265	0.025
$C_{11}H_{16}O_3$	127657-97-0	0.14 ± 0.02	0.140	0.279	0.015
$C_{13}H_{21}NO_4$	1000328-61-7	0.14 ± 0.02	0.138	0.276	0.019
$C_{27}H_{55}NO_2$	1000415-18-9	0.14 ± 0.02	0.137	0.275	0.031
$C_{17}H_{16}O_4$	1775-97-9	0.14 ± 0.02	0.136	0.272	0.021
$C_{14}H_{13}N_3OS_2$	155670-84-1	0.14 ± 0.02	0.140	0.281	0.022
$C_{12}H_{15}NO_2S$	128869-50-1	0.13 ± 0.21	0.063	1.028	0.018
$C_{16}H_{22}N_4O_9$	41545-25-9	0.13 ± 0.20	0.045	0.848	0.030
$C_{16}H_8ClNO_2$	138793-86-9	0.13 ± 0.13	0.073	0.578	0.021
$C_{17}H_{14}O_4$	55927-39-4	0.13 ± 0.12	0.079	0.480	0.021
$C_9H_9N_7O_3$	1000443-60-2	0.13 ± 0.08	0.096	0.371	0.020
$C_{10}H_{17}N_5$	164589-37-1	0.13 ± 0.08	0.102	0.403	0.016
$C_{15}H_{15}N_3O_3$	1000350-12-2	0.13 ± 0.07	0.103	0.319	0.021
$C_{13}H_8F_3NO_4$	1000397-48-3	0.13 ± 0.07	0.114	0.407	0.022
$C_9H_8N_2O_2S$	23766-26-9	0.13 ± 0.06	0.113	0.349	0.016

$C_{12}H_{18}O_2Si$	33342-87-9	0.13 ± 0.05	0.110	0.336	0.017
$C_5H_8O_3$	3952-66-7	0.13 ± 0.05	0.122	0.318	0.009
$C_{10}H_{18}O_2$	103619-06-3	0.13 ± 0.04	0.123	0.253	0.013
$C_{18}H_{19}NO_2$	52119-37-6	0.13 ± 0.04	0.123	0.247	0.021
$C_{10}H_{11}ClO$	2623-45-2	0.13 ± 0.03	0.122	0.244	0.014

Table S1 (cont.). Basic characteristics of chemical components in indoor $PM_{2.5}$ (ng/m^3)

**Components**	**CAS Number**	**Mean ± SD**	**Minimum**	**Maximum**	**LOD （ng/m^3）**
$C_{20}H_{25}NO_4$	1000398-24-5	0.13 ± 0.03	0.124	0.248	0.025
$C_9H_9IN_6$	1000303-11-3	0.13 ± 0.03	0.121	0.242	0.024
$C_{18}H_{19}NO$	1000401-64-0	0.13 ± 0.03	0.121	0.242	0.020
$C_8H_6O_2$	623-27-8	0.13 ± 0.03	0.121	0.242	0.010

Note: LOD refers to the limit of detection and is expressed in ng/m^3. All LOD values in this table are retained to three decimal places.

Table S2. Basic characteristics of chemical components in individual $PM_{2.5}$ (ng/m^3)

**Components**	**CAS Number**	**Mean ± SD**	**Minimum**	**Maximum**	**LOD （ng/m^3）**
$C_5H_{12}N_2O_2S$	1000303-61-8	4.31 ± 0.38	4.273	8.547	0.013
$C_8H_9N_7O_3$	1000436-20-7	4.31 ± 0.38	4.275	8.550	0.019
C_6H_4BrClO	695-96-5	4.31 ± 0.38	4.282	8.563	0.016
$C_{10}H_{11}ClO_3$	1000365-10-3	4.30 ± 0.38	4.268	8.536	0.016
$C_{21}H_{17}NO_3$	339229-39-9	4.30 ± 36.93	0.635	420.115	0.024
$C_{12}H_{24}$	63830-67-1	4.30 ± 0.38	4.266	8.533	0.013
$C_{11}H_{13}NO_3$	1000307-09-1	4.30 ± 12.44	1.810	121.443	0.016
$C_{17}H_{14}N_2O_5$	329932-15-2	4.29 ± 0.37	4.255	8.510	0.024
$C_2H_4Cl_2O_4S_2$	31469-08-6	4.29 ± 0.37	4.252	8.504	0.017
$C_9H_{19}N$	108144-19-0	4.29 ± 0.37	4.258	8.517	0.011
$C_{14}H_{28}O_2Si$	1000421-14-1	4.29 ± 0.38	4.262	8.523	0.019
$C_4H_6O_2$	1759-53-1	4.29 ± 0.37	4.257	8.513	0.007
C_9H_{18}	26456-76-8	4.29 ± 0.37	4.258	8.515	0.010
$C_{15}H_{12}O$	32555-96-7	4.29 ± 0.37	4.256	8.513	0.016
$C_{15}H_{26}O_5$	1000390-71-5	4.28 ± 0.37	4.247	8.494	0.021
$C_{14}H_{26}$	3321-50-4	4.28 ± 0.37	4.249	8.497	0.015
$C_{12}H_{14}O_4$	84-66-2	4.27 ± 13.66	0.770	127.585	0.017
$C_{18}H_{18}O_3$	1000449-91-2	4.27 ± 0.37	4.234	8.469	0.021
$C_6H_{14}N_2O_2$	4164-29-8	4.27 ± 0.37	4.239	8.477	0.011
$C_9H_{14}O_3$	68133-76-6	4.27 ± 0.37	4.241	8.483	0.013
$C_9H_{16}O$	104188-14-9	4.27 ± 0.37	4.235	8.470	0.011

$C_{25}H_{49}NO_5$	1000340-12-8	4.27 ± 0.37	4.242	8.483	0.032
$C_{12}H_{18}O_4$	13048-33-4	4.26 ± 17.97	1.824	200.489	0.017
$C_{17}H_{35}NO$	6280-57-5	4.26 ± 0.37	4.223	8.445	0.020
$C_7H_7ClN_4O$	6504-64-9	4.26 ± 0.37	4.227	8.453	0.015
$C_7H_{13}N_3$	23974-29-0	4.26 ± 0.37	4.226	8.452	0.011
$C_{21}H_{44}O$	1000406-37-5	4.25 ± 0.37	4.214	8.429	0.023
$C_{22}H_{42}F_3NO_4Si_4$	1000072-26-7	4.24 ± 0.37	4.207	8.413	0.040
C_5H_7NS	27330-47-8	4.24 ± 0.37	4.206	8.412	0.009
$C_{14}H_8N_4S_2$	54255-48-0	4.24 ± 0.37	4.207	8.414	0.022
C_8H_8O	98-86-2	4.24 ± 1.92	3.949	22.720	0.010

Table S2 (cont.). Basic characteristics of chemical components in individual $PM_{2.5}$ (ng/m^3)

Components	CAS Number	Mean ± SD	Minimum	Maximum	LOD （ng/m^3）
$C_3H_6N_4$	1185158	4.23 ± 16.90	2.552	193.794	0.008
$C_{16}H_{14}N_2OS$	6825-98-5	4.23 ± 0.37	4.193	8.387	0.021
$C_7H_{15}N_3O_2$	50285-70-6	4.22 ± 0.37	4.191	8.382	0.013
$C_{14}H_{12}O_2$	1000279-96-1	4.22 ± 0.37	4.191	8.382	0.016
$C_{18}H_{20}$	110551-72-9	4.22 ± 0.37	4.190	8.380	0.018
$C_{11}H_{22}O$	1000432-13-0	4.22 ± 0.37	4.190	8.380	0.013
$C_{11}H_{13}F_7O_4$	1000365-43-2	4.21 ± 0.37	4.180	8.360	0.025
$C_{15}H_{18}O_5$	1000314-91-3	4.21 ± 0.37	4.178	8.356	0.021
$C_7H_{16}O$	27522-11-8	4.20 ± 0.37	4.164	8.328	0.009
$C_3H_5N_5O$	328977-78-2	4.20 ± 0.37	4.172	8.345	0.010
$C_{13}H_{19}NO_4Si$	55638-48-7	4.20 ± 0.37	4.167	8.334	0.021
$C_{27}H_{42}O_4$	1000314-92-1	4.20 ± 0.37	4.165	8.329	0.031
$C_6H_{14}O_3$	77-99-6	4.19 ± 0.37	4.154	8.308	0.010
$C_{16}H_{11}N$	2693-46-1	4.19 ± 0.37	4.160	8.320	0.016
$C_{15}H_{12}N_2O_2$	25470-39-7	4.19 ± 19.12	1.083	213.841	0.019
$C_{10}H_{18}O_2$	16491-36-4	4.19 ± 0.37	4.156	8.313	0.013
$C_{19}H_{18}F_2O_6$	1000393-57-2	4.19 ± 0.52	4.121	8.322	0.028
C_8H_{16}	16746-86-4	4.18 ± 0.37	4.152	8.305	0.009
$C_{14}H_{18}O_6$	3044-56-2	4.18 ± 0.37	4.149	8.298	0.021
$C_{16}H_{24}F_6O_4$	1000390-80-5	4.17 ± 0.36	4.133	8.266	0.029
$C_{13}H_9NO_2$	607-57-8	4.16 ± 0.36	4.133	8.265	0.016
$C_8H_{12}O_3$	2399-48-6	4.16 ± 0.36	4.130	8.261	0.012
$C_{11}H_{15}ClN_4O$	1000263-69-5	4.15 ± 0.36	4.119	8.238	0.019
$C_{15}H_{13}F_7O_4$	1000357-53-1	4.14 ± 6.73	3.305	69.769	0.029
C_9H_{18}	13151-06-9	4.13 ± 0.36	4.102	8.205	0.010
$C_{14}H_{28}$	62376-15-2	4.13 ± 0.36	4.097	8.193	0.015
$C_8H_8N_2O$	72716-80-4	4.12 ± 0.36	4.089	8.179	0.011
$C_{10}H_{18}O_2$	34426	4.11 ± 0.36	4.074	8.147	0.013

$C_{12}H_{16}O_3$	1000330-12-4	4.11 ± 0.36	4.081	8.161	0.016
$C_5H_5NO_4S_2$	1000300-36-6	4.11 ± 16.28	2.154	179.630	0.016
$C_7H_4N_4O_4$	31208-76-1	4.11 ± 0.36	4.080	8.160	0.016
$C_{14}H_{11}N_3O_2$	18450-09-4	4.11 ± 0.54	4.041	8.613	0.019
$C_{13}H_{28}O$	85763-57-1	4.10 ± 0.36	4.070	8.140	0.015
$C_{17}H_{30}O_4$	1000391-31-7	4.10 ± 0.36	4.065	8.129	0.022
$C_{12}H_{11}F_3N_2O_5$	96760-73-5	4.10 ± 6.62	2.465	53.195	0.024
$C_{19}H_{23}NO$	1000446-79-6	4.09 ± 28.26	1.040	321.278	0.021
$C_{15}H_9NO_4$	1000401-67-0	4.09 ± 0.36	4.063	8.126	0.020
$C_9H_7NO_4S$	83039-60-5	4.09 ± 0.36	4.058	8.117	0.017
$C_4H_7BrO_2$	4360-63-8	4.09 ± 0.36	4.055	8.110	0.013
$C_{11}H_8N_2$	233-53-4	4.09 ± 0.36	4.056	8.111	0.013

Table S2 (cont.). Basic characteristics of chemical components in individual $PM_{2.5}$ (ng/m^3)

**Components**	**CAS Number**	**Mean ± SD**	**Minimum**	**Maximum**	**LOD （ng/m³）**
$C_9H_6ClF_9O_4$	1000375-55-7	4.08 ± 0.36	4.046	8.092	0.028
$C_{15}H_{13}N$	30020-98-5	4.08 ± 8.53	2.227	60.725	0.016
$C_{12}H_{17}NO$	1000163-19-5	4.08 ± 0.36	4.051	8.103	0.015
$C_{17}H_{19}N_3O$	1000302-50-5	4.07 ± 7.38	2.343	59.650	0.021
$C_{32}H_{57}F_7O_2$	1000351-83-6	4.07 ± 0.36	4.040	8.080	0.044
$C_{10}H_{11}N_3O_3S$	1000396-68-3	4.07 ± 0.36	4.036	8.071	0.019
$C_{16}H_{22}N_4O_9$	41545-25-9	4.07 ± 11.96	0.306	68.846	0.030
$C_{15}H_{25}NO_4$	1000325-14-3	4.07 ± 0.36	4.037	8.075	0.021
$C_{19}H_{20}O_4$	1000356-76-0	4.07 ± 0.36	4.040	8.080	0.023
$C_{23}H_{18}O_2$	1000402-36-9	4.07 ± 4.29	3.186	41.507	0.024
$C_8H_6F_3NO_4S$	1000302-85-5	4.07 ± 2.19	3.850	28.443	0.020
$C_{14}H_{21}NO_2$	1000446-72-3	4.06 ± 0.35	4.031	8.062	0.018
$C_{13}H_{24}O_2$	1000221-98-6	4.06 ± 0.35	4.027	8.055	0.016
$C_{14}H_{28}N_6$	19455-91-5	4.06 ± 0.36	4.032	8.064	0.021
$C_5H_6F_6S$	380-35-8	4.06 ± 0.35	4.028	8.056	0.016
$C_{14}H_{15}N_3O_2$	117846-36-3	4.06 ± 0.36	4.033	8.066	0.019
$C_{21}H_{40}O_4$	1000363-93-6	4.06 ± 0.35	4.027	8.053	0.026
$C_{13}H_{18}O_2$	5406-57-5	4.05 ± 35.09	0.745	398.583	0.016
$C_3H_6N_2O$	15216-10-1	4.05 ± 0.35	4.019	8.038	0.007
$C_9H_{16}O_2$	1000109-76-5	4.05 ± 0.35	4.021	8.042	0.012
$C_{18}H_{39}BO_3$	5337-36-0	4.04 ± 0.35	4.005	8.010	0.023
$C_6H_{11}NO$	931-20-4	4.04 ± 0.35	4.011	8.021	0.009
$C_{13}H_{22}O_5$	1000330-64-3	4.04 ± 0.35	4.007	8.013	0.019
$C_{13}H_{11}N_7O$	311783-53-6	4.04 ± 0.35	4.007	8.013	0.021
$C_5H_8O_2$	108-22-5	4.03 ± 0.35	3.994	7.988	0.008
$C_{13}H_{16}N_2O$	1000302-78-5	4.03 ± 0.35	3.994	7.988	0.016

$C_{18}H_{26}O_4$	131-18-0	4.03 ± 0.35	4.001	8.002	0.023
$C_{19}H_{13}N_5$	1000327-04-1	4.03 ± 11.05	1.196	109.611	0.023
$C_{14}H_{13}F_5O_4$	1000415-77-4	4.03 ± 0.35	4.003	8.005	0.025
$C_7H_6N_4O_2$	6726-55-2	4.03 ± 0.35	3.995	7.991	0.014
$C_{13}H_{16}O_3$	1000453-46-1	4.02 ± 0.35	3.988	7.976	0.017
$C_{10}H_{11}ClO_2$	63867-11-8	4.02 ± 0.35	3.992	7.984	0.015
$C_{14}H_{18}O_3$	49763-96-4	4.02 ± 0.35	3.984	7.968	0.018
$C_{19}H_{40}O_2$	1000406-35-2	4.02 ± 0.35	3.990	7.981	0.022
$C_{10}H_{10}O_4$	1000241-63-9	4.01 ± 0.35	3.979	7.957	0.015
C_9H_9ClO	936-59-4	4.01 ± 0.35	3.979	7.958	0.013
$C_9H_{11}NO_2$	1129-41-5	4.01 ± 0.35	3.977	7.955	0.013
CF_3I	2314-97-8	4.00 ± 0.35	3.968	7.937	0.015
$C_{12}H_{29}O_3PSi$	1000383-80-7	4.00 ± 0.35	3.965	7.930	0.021
$C_{14}H_{12}N_2O_3$	15450-66-5	4.00 ± 0.35	3.974	7.947	0.019

Table S2 (cont.). Basic characteristics of chemical components in individual $PM_{2.5}$ (ng/m^3)

**Components**	**CAS Number**	**Mean ± SD**	**Minimum**	**Maximum**	**LOD （ng/m^3）**
$C_{14}H_{24}N_2$	101-96-2	3.99 ± 0.35	3.961	7.922	0.017
$C_4H_{10}N_2$	113604-56-1	3.99 ± 0.35	3.962	7.924	0.007
$C_{36}H_{70}O_5Si_4$	1000418-96-1	3.99 ± 0.35	3.963	7.925	0.050
$C_{18}H_{38}O$	110225-00-8	3.99 ± 0.35	3.960	7.920	0.020
$C_{15}H_{29}NO$	1000458-92-1	3.99 ± 17.74	1.583	197.181	0.018
$C_8H_{14}N_4$	13717-92-5	3.98 ± 0.35	3.954	7.908	0.013
$C_{24}H_{54}O_2Si_2$	1000336-70-6	3.98 ± 0.35	3.951	7.902	0.031
$C_{10}H_{18}O_3$	78641-04-0	3.98 ± 0.35	3.945	7.890	0.014
$C_{10}H_{22}O_2$	1559-35-9	3.98 ± 0.35	3.951	7.901	0.013
$C_{19}H_{40}O_3S$	1000309-13-5	3.98 ± 2.86	3.615	32.059	0.026
$C_{16}H_{16}O_2$	1000293-61-2	3.97 ± 0.35	3.936	7.872	0.018
$C_{11}H_{14}N_2O_3S$	314765-48-5	3.97 ± 0.35	3.936	7.873	0.019
$C_8H_{10}N_2$	18217-81-7	3.97 ± 0.35	3.939	7.877	0.010
$C_2H_4N_2$	540-61-4	3.96 ± 38.17	0.536	434.048	0.005
C_6H_{12}	96-37-7	3.96 ± 0.35	3.929	7.857	0.007
$C_{21}H_{30}O_4$	1000344-95-3	3.95 ± 0.34	3.918	7.836	0.025
$C_9H_{14}O$	17587-33-6	3.95 ± 0.34	3.915	7.831	0.011
$C_9H_{18}O$	34061-78-4	3.94 ± 0.34	3.906	7.811	0.011
$C_6H_5N_3O_2$	65996-50-1	3.94 ± 23.21	0.226	251.952	0.012
$C_2H_2N_4O_2$	24807-55-4	3.94 ± 4.80	2.407	35.075	0.009
$C_{11}H_{24}$	62016-33-5	3.93 ± 16.06	2.187	183.744	0.012
$C_4H_6BrN_3$	1000459-77-6	3.92 ± 0.34	3.888	7.777	0.013
$C_7H_{10}O_2$	112595-65-0	3.92 ± 0.34	3.892	7.785	0.010
$C_{12}H_{18}O_2$	53847-40-8	3.92 ± 0.34	3.888	7.777	0.015

$C_{19}H_{28}O_5S$	1000160-04-2	3.92 ± 0.34	3.894	7.788	0.027
$C_{16}H_{14}OS$	79134-84-2	3.91 ± 1.51	3.755	20.549	0.019
$C_{27}H_{36}Cl_2N_4O_3$	1000285-78-9	3.91 ± 0.34	3.876	7.753	0.039
$C_2HCl_2F_3$	354-23-4	3.90 ± 0.34	3.872	7.744	0.012
$C_{15}H_7NO_3S$	1000303-19-5	3.90 ± 2.93	3.322	27.993	0.021
$C_9H_{13}F_5O_2$	1000352-35-9	3.89 ± 0.34	3.855	7.710	0.019
$C_7H_{16}O$	13231-81-7	3.89 ± 0.34	3.864	7.728	0.009
$C_{11}H_6ClNO_4$	1000254-68-4	3.88 ± 11.88	2.072	110.907	0.019
$C_{24}H_{41}NO_4$	1000383-19-2	3.88 ± 0.34	3.846	7.691	0.030
$C_{22}H_{15}NOS_2$	1000296-76-2	3.88 ± 0.34	3.848	7.696	0.027
C_2H_4ClN	10165-13-6	3.88 ± 0.34	3.845	7.691	0.007
$C_{16}H_{24}O_2$	104316-22-5	3.87 ± 0.34	3.842	7.684	0.019
$C_8H_{18}O$	104-76-7	3.86 ± 9.66	0.699	52.596	0.010
$C_{22}H_{46}O_2Si$	1000363-55-6	3.86 ± 0.34	3.828	7.657	0.027
$C_4H_4O_4$	502-97-6	3.86 ± 0.34	3.834	7.667	0.009
$C_2H_4N_4O_2$	21531-96-4	3.86 ± 8.09	2.795	92.771	0.009

Table S2 (cont.). Basic characteristics of chemical components in individual $PM_{2.5}$ (ng/m^3)

Components	CAS Number	Mean ± SD	Minimum	Maximum	LOD （ng/m^3）
$C_{14}H_{20}N_2O$	1982-49-6	3.85 ± 0.34	3.823	7.646	0.017
$C_{17}H_{17}NO_3$	128944-43-4	3.85 ± 0.34	3.824	7.647	0.021
$C_{10}H_{22}OSi$	1000352-68-8	3.84 ± 0.34	3.813	7.627	0.014
$C_{12}H_{22}O_4$	1000349-44-0	3.84 ± 0.34	3.808	7.617	0.017
$C_{12}H_{11}NO_3$	15495-17-7	3.84 ± 0.34	3.807	7.614	0.016
$C_{10}H_{17}N_5$	164589-37-1	3.84 ± 8.12	1.694	73.747	0.016
$C_{13}H_{17}NO_4$	1000120-28-7	3.84 ± 0.34	3.813	7.625	0.019
$C_6H_8O_3$	517-23-7	3.83 ± 0.33	3.799	7.598	0.010
$C_{18}H_{19}NO$	1000300-98-6	3.83 ± 0.33	3.802	7.605	0.020
$C_{19}H_{14}N_4O$	1000317-39-5	3.83 ± 0.33	3.801	7.603	0.023
$C_{18}H_{16}O_5$	1000443-99-9	3.82 ± 12.84	1.388	108.739	0.023
$C_{14}H_9F_2NO_4$	1000357-68-7	3.81 ± 0.33	3.778	7.556	0.022
$C_{10}H_{22}Si$	1000427-30-8	3.81 ± 0.47	3.752	7.504	0.013
$C_{13}H_{15}NO_4$	1000406-90-6	3.80 ± 0.33	3.770	7.540	0.019
$C_{11}H_{27}NSi_3$	7351-44-2	3.79 ± 0.33	3.764	7.529	0.019
$C_{14}H_{10}O$	90-44-8	3.78 ± 0.33	3.746	7.492	0.015
$C_{14}H_{22}O_3Si$	1000352-92-3	3.78 ± 0.33	3.747	7.494	0.020
$C_{12}H_{10}N_2O_2$	91973-67-0	3.78 ± 0.33	3.750	7.499	0.016
$C_{16}H_{18}O_4S$	3795-83-3	3.78 ± 0.33	3.750	7.499	0.023
$C_{22}H_{17}NO_4$	1000304-79-3	3.78 ± 0.76	3.696	11.514	0.026
$C_5H_7BrO_2$	99393-06-3	3.78 ± 0.33	3.748	7.496	0.014
$C_8H_{15}NO_4$	1603543	3.77 ± 0.33	3.738	7.476	0.014

$C_{11}H_{11}BrO$	1000443-02-2	3.77 ± 0.79	3.634	9.375	0.018
$C_7H_{14}N_2O_3$	1000375-78-2	3.76 ± 0.33	3.730	7.461	0.013
$C_4H_8N_2$	592-56-3	3.76 ± 0.33	3.730	7.460	0.007
$C_8H_{12}O_2$	1000136-98-1	3.76 ± 0.33	3.727	7.455	0.011
$C_{17}H_{34}$	41977-41-7	3.76 ± 0.33	3.729	7.457	0.018
$C_{13}H_{10}N_2O$	75204-58-9	3.76 ± 0.33	3.734	7.467	0.016
$C_{14}H_{20}O_2S$	1000278-96-1	3.75 ± 0.33	3.722	7.444	0.019
$C_5H_6O_2$	627-09-8	3.75 ± 0.33	3.721	7.443	0.008
$C_{17}H_{31}Cl_3O_2$	74339-53-0	3.75 ± 0.33	3.717	7.433	0.027
$C_{12}H_{18}O$	107081-99-2	3.75 ± 0.33	3.725	7.450	0.014
$C_{14}H_{26}O_2Si_2$	36461-33-3	3.75 ± 23.29	0.351	257.350	0.021
$C_{10}H_8F_4O$	207974-18-3	3.74 ± 0.33	3.709	7.418	0.017
$C_4H_8O_3$	623-53-0	3.74 ± 0.33	3.709	7.417	0.008
C_3H_9Al	75-24-1	3.74 ± 0.33	3.712	7.424	0.006
$C_{12}H_{17}NO_2$	1000339-98-1	3.73 ± 7.41	1.785	44.427	0.016
$C_{10}H_{11}NO_3S$	1000303-70-7	3.73 ± 0.33	3.701	7.401	0.017
$C_6H_9NO_2$	15731-98-3	3.73 ± 0.33	3.702	7.404	0.010
$C_{15}H_{24}N_2O_5$	1000434-15-3	3.73 ± 0.33	3.704	7.409	0.023

Table S2 (cont.). Basic characteristics of chemical components in individual $PM_{2.5}$ (ng/m^3)

**Components**	**CAS Number**	**Mean ± SD**	**Minimum**	**Maximum**	**LOD （ng/m^3）**
$C_{19}H_{20}O_6$	1000344-53-1	3.73 ± 0.33	3.705	7.410	0.025
$C_{14}H_{12}O_2$	1000426-41-8	3.72 ± 0.33	3.693	7.387	0.016
$C_{14}H_9FN_2O$	330682-78-5	3.72 ± 0.33	3.695	7.390	0.018
$C_{20}H_{15}N_3O_2$	1000317-39-7	3.72 ± 0.33	3.693	7.387	0.024
$C_5H_{13}B_3O_3$	1000162-61-0	3.71 ± 0.32	3.682	7.364	0.012
$C_{11}H_{17}NO$	135014-85-6	3.71 ± 0.32	3.682	7.364	0.014
$C_{14}H_{30}O$	4706-81-4	3.71 ± 0.32	3.681	7.362	0.016
$C_7H_{14}O_5$	36680-67-8	3.71 ± 0.32	3.679	7.359	0.014

Note: LOD refers to the limit of detection and is expressed in ng/m^3. All LOD values in this table are retained to three decimal places.

Table S3. Wilcoxon signed-rank test comparing individual and indoor $PM_{2.5}$ concentrations

**Comparison**	**Total**	**Negative Ranks**			**Positive Ranks**			**Z**	**P（2-tailed)**
		N	Mean Rank	Sum of Rank	N	Mean Rank	Sum of Rank		
Individual vs Indoor	258	43	56.44	2427.00	215	144.11	30984.00	-11.90	<0.001

Table S4. T-test of the difference (individual – outdoor) in $PM_{2.5}$ concentrations

**Variable**	**N**	**Mean**	**Std.Dev.**	**Std.Error Mean**	**t**	**df**	**p（2-tailed)**	**Mean Difference**	**95% Cl**
difference	258	7.869	48.701	3.0324	2.595	257	0.010	7.869	1.896-13.841
