# Supplementary material for: From Environmental Concentrations to Individual Inhalation: Analysis of Exposure Differences to PM2.5 and Chemical Components in Elderly Populations and Their Influencing Factors
Source: Toxics. 2026 May 10;14(5):414. doi: 10.3390/toxics14050414 (PMC13211666; doi:10.3390/toxics14050414)
Supplement: Supplementary file 1 [file toxics-14-00414-s001.zip › Box plot.pdf]

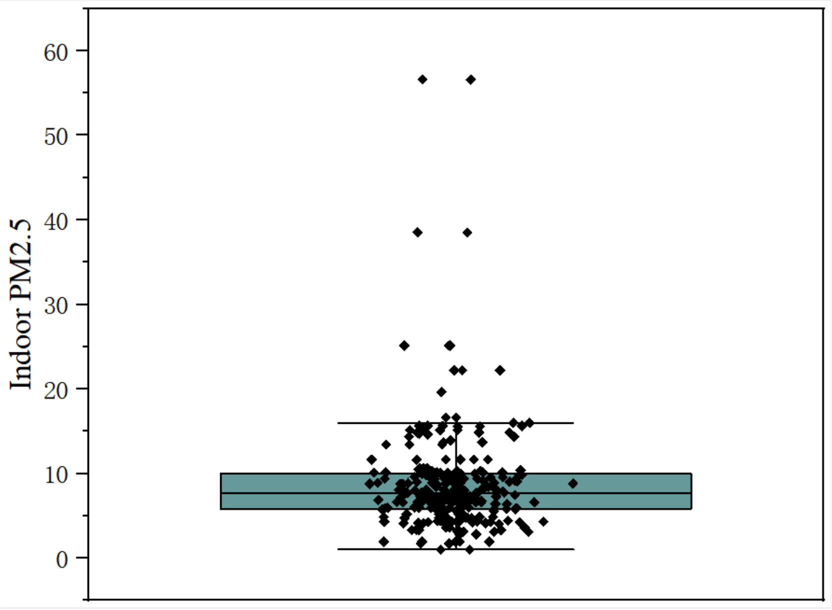

Figure S1. Indoor $PM_{2.5}$ concentrations across study participants

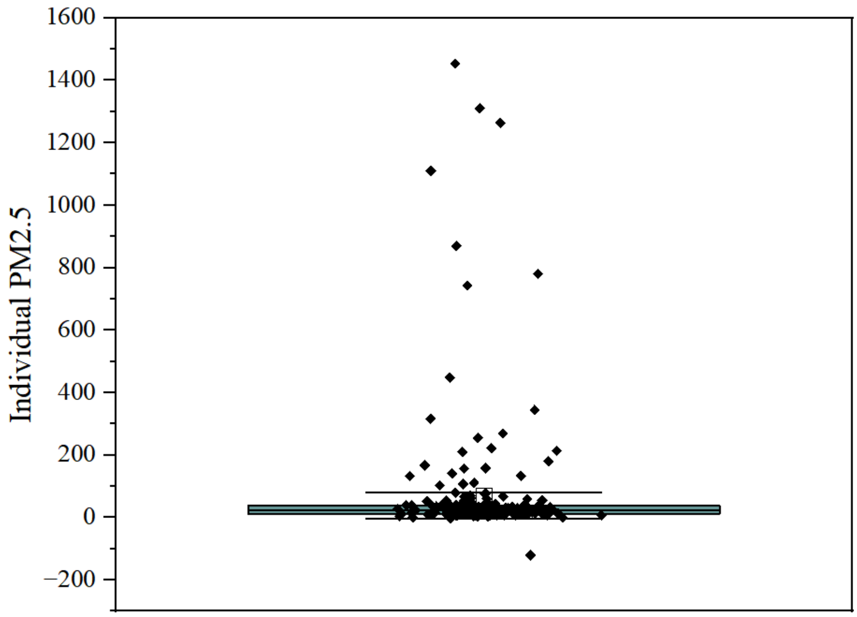

Figure S2. Individual $PM_{2.5}$ exposure concentrations across study participants
